# The Insertion Green Monster (iGM) Method for Expression of Multiple Exogenous Genes in Yeast

**DOI:** 10.1534/g3.114.010868

**Published:** 2014-04-28

**Authors:** Vyacheslav M. Labunskyy, Yo Suzuki, Timothy J. Hanly, Ayako Murao, Frederick P. Roth, Vadim N. Gladyshev

**Affiliations:** * Division of Genetics, Department of Medicine, Brigham and Women’s Hospital and Harvard Medical School, Boston, Massachusetts 02115; † Department of Synthetic Biology and Bioenergy, J. Craig Venter Institute, La Jolla, California 92037; ‡ Donnelly Centre and Departments of Molecular Genetics and Computer Science, University of Toronto, Toronto, Ontario M5S 3E1, Canada; § Lunenfeld-Tanenbaum Research Institute, Mt. Sinai Hospital, Toronto, Ontario M5T 3L9, Canada

**Keywords:** synthetic biology, multi-gene insertions, *Saccharomyces cerevisiae*, green fluorescent protein, flow cytometry

## Abstract

Being a simple eukaryotic organism, *Saccharomyces cerevisiae* provides numerous advantages for expression and functional characterization of proteins from higher eukaryotes, including humans. However, studies of complex exogenous pathways using yeast as a host have been hampered by the lack of tools to engineer strains expressing a large number of genetic components. In addition to inserting multiple genes, it is often desirable to knock out or replace multiple endogenous genes that might interfere with the processes studied. Here, we describe the “insertion Green Monster” (iGM) set of expression vectors that enable precise insertion of many heterologous genes into the yeast genome in a rapid and reproducible manner and permit simultaneous replacement of selected yeast genes. As a proof of principle, we have used the iGM method to replace components of the yeast pathway for methionine sulfoxide reduction with genes encoding the human selenoprotein biosynthesis machinery and generated a single yeast strain carrying 11 exogenous components of the selenoprotein biosynthetic pathway in precisely engineered loci.

The yeast *Saccharomyces cerevisiae* is a simple model organism for which extensive resources have been generated for forward and reverse genetics and functional and phenotypic characterization. Due to its high conservation of core processes shared with higher eukaryotes, yeast can provide a relevant cellular environment for the characterization of exogenous pathways, for example, of human origin, when confounding activities are removed in yeast via evolution or genetic engineering. Recent advances in systems biology and genome-manipulation technologies have dramatically expanded our ability to engineer cells in a directed manner. However, characterization of complex human pathways in yeast has been challenging due to a shortage of tools to express more than a few exogenous genes. A commonly used procedure involves generating a large plasmid containing many genes of interest (Hamilton *et al.* 2006) or breaking-up the genes into several plasmids, each containing one or a few genes (Engels *et al.* 2008). Constructing a large plasmid is not always straightforward, and parts of the resulting plasmid are often not readily exchangeable. The strategy involving separate plasmids addresses these problems, but the number of available selectable markers or compatible plasmid systems is insufficient for studying complex pathways. This approach also suffers from the difficulty of achieving precise expression levels for gene products because copy numbers of plasmids are variable.

Selenocysteine (Sec) is an unusual amino acid encoded by the UGA codon (Hatfield and Gladyshev 2002). It is present in the active sites of selenium-containing proteins and is co-translationally inserted into nascent polypeptides through mechanisms distinct from normal protein synthesis (Driscoll and Copeland 2003). This process requires special protein factors, a Sec transfer RNA (Sec tRNA) and a Sec insertion sequence (SECIS) element in the 3′-untranslated region of selenoprotein mRNA. Whereas Sec biosynthesis and insertion pathways are conserved in diverse eukaryotic lineages, they have been lost in fungi. Reconstitution of Sec insertion has not been previously reported, and currently it is not known whether introduction of human factors into *S. cerevisiae* is sufficient. Therefore, the budding yeast may provide an ideal test-bed for the functional characterization of the Sec pathway.

The aim of our current study was to develop a new technology to create yeast strains carrying multiple exogenous genes by integrating them into precisely engineered loci in the yeast genome. To this end, we have adapted a recently described strategy (the “Green Monster” method) that allows the assembly of multiple engineered loci, each marked by a quantitatively selectable green fluorescent protein (GFP) marker, into a single strain (Suzuki *et al.* 2011, 2012). The Green Monster process was previously used to delete multiple genes within a given strain, but not to assemble multiple loci each containing an exogenous gene. Here, we extend the versatility of this method by applying it to the engineering of gene insertions. We have found that the new insertion Green Monster (iGM) approach is particularly useful for the characterization of multiple genes of a complex exogenous pathway in a stepwise fashion, by virtue of the placement of the introduced genes at unlinked loci in *S. cerevisiae*. In a proof-of-concept experiment, we have used the iGM method to assemble 11 exogenous genes encoding components of the metazoan Sec biosynthesis and insertion pathways into a single strain.

## Materials and Methods

### Yeast strains and media

The yeast strains used in this study were from the Yeast Knockout Collection (Giaever *et al.* 2002) or were derived more directly from BY4741 (*MAT***a** *his3Δ1 leu2Δ0 met15Δ0 ura3Δ0*) or BY4742 (*MATα his3Δ1 leu2Δ0 lys2Δ0 ura3Δ0*). The GMToolkit-**a** (RY0146) and GMToolkit-α (RY0148) strains have the background genotype *MAT*α *lyp1Δ his3Δ1 leu2Δ0 ura3Δ0 met15Δ0* and have *can1Δ*::*GMToolkit-***a** (*CMVpr-rtTA KanMX4 STE2pr-Sp-his5*) and *can1Δ*::*GMToolkit-α* (*CMVpr-rtTA NatMX4 STE3pr-LEU2*), respectively (Suzuki *et al.* 2011). The genotype of iGM6 (LY0155) and iGM11 (LY0156) insertion monsters are *MAT***a** *ykl069wΔ*::*TRSP yer042wΔ*::*PSTK ycl033cΔ*::*SBP2 yol118cΔ*::*SECS ydl242wΔ*::*EEFSEC ydl227cΔ*::*cSPS2 can1Δ*::*GMToolkit-***a** *trp1Δ*::*GPDpr-MsrA(C72U)-SECIS-HphMX4 his3Δ1 leu2Δ0 met15Δ0 ura3Δ0* and *MAT***a** *ykl069wΔ*::*TRSP yer042wΔ*::*PSTK ycl033cΔ*::*SBP2 yol118cΔ*::*SECS ydl242wΔ*::*EEFSEC ydl227cΔ*::*cSPS2 ylr123cΔ*::*SPS1 yer108c Δ*::*SPS2 ygl109wΔ*::*SECP43 yfr057wΔ*::*RPL30 ykr011cΔ*::*SBP2L can1Δ*::*GMToolkit*-**a** *trp1Δ*::*GPDpr-MsrA(C72U)-SECIS-HphMX4 his3Δ1 leu2Δ0 met15Δ0 ura3Δ0*, respectively. Each insertion locus also contains the *GFP* reporter gene *ADHterm-tetO_2_pr-GFP*(S65T)-*CYC1term* and the transformation marker *URA3* as part of the universal gene insertion module (see below). All yeast strains were grown on complete YPD medium (1.0% yeast extract, 2.0% peptone, and 2.0% glucose) or on synthetic complete (SC) medium lacking the relevant auxotrophic component, unless otherwise noted.

### Generation of the universal GFP cassette for gene insertion

**Figure 1:**
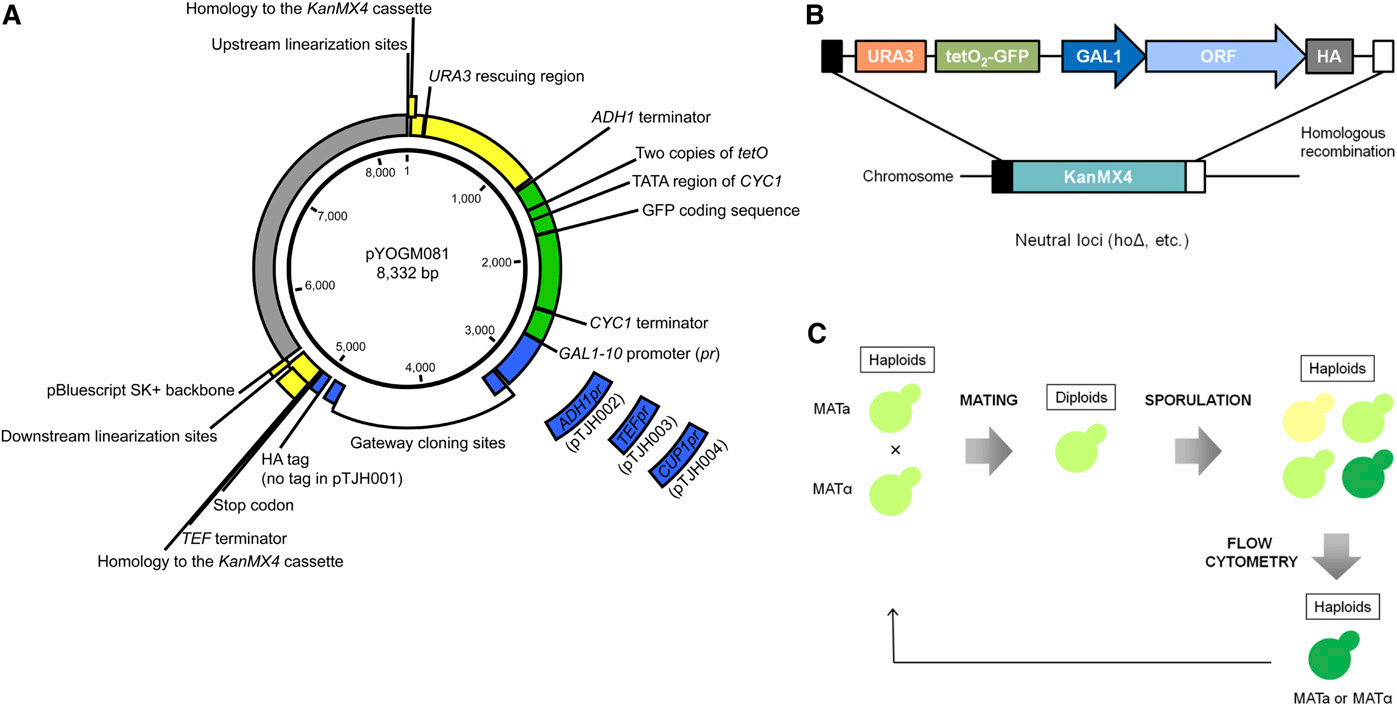
The insertion Green Monster (iGM) strategy. (A) Schematic map of the iGM expression vectors. (B) A universal GFP insertion cassette containing the exogenous gene under control of the *GAL1-10* promoter. Each gene is cloned into the insertion cassette using the Gateway cloning reaction such that it contains a C-terminal hemagglutinin (HA) epitope tag. The insertion cassette replaces *KanMX4* in a neutral locus via homologous recombination. (C) Schematic overview of the Green Monster process for sexual cycling and enrichment of strains containing multiple *GFP* copies using flow cytometry.

The gene insertion module is harbored in the plasmid pYOGM081 (Figure 1A and Supporting Information, Figure S1). It contains sequences homologous to the ends of the *KanMX4* marker for gene targeting (Wach *et al.* 1994) flanking a *URA3* transformation marker, a *GFP* reporter gene, and a set of sequences enabling the overexpression of a gene of interest. Eight restriction sites (*Nhe*I, *Sac*II, *Sna*BI, *Sex*AI, *Sac*I, *Bgl*II, *Pac*I, and *Asc*I) are present on either side of the gene insertion module so that one or more of the corresponding restriction enzymes would be available for creating free ends that enhance homologous recombination without destroying the insertion module for many genes. The *GFP* reporter gene contains the transcriptional terminator from the *ADH* gene, duplicated *tet* operator (*tetO_2_*), the GFP coding sequence *GFP(S65T)*, and the terminator from the *CYC1* gene, in this order (Suzuki *et al.* 2011). The sequences for overexpression were derived from the plasmid pAG416GAL-ccdB-HA (Alberti *et al.* 2007). They include the inducible promoter *GAL1-10pr*, a Gateway-compatible cloning site (Life Technologies), a sequence for the HA-tag, and a stop codon. The *CYC1* terminator used in pAG416GAL-ccdB-HA is replaced with the *TEF* terminator (Wach *et al.* 1994) in pYOGM081. To generate pYOGM081, five fragments were sequentially introduced into the plasmid pBluescript SK+. First, a 0.3-kb PCR fragment containing the terminator of *KanMX4* and restriction sites for linearization (fragment 1; Table S1) was digested using the enzymes *Hin*dIII and *Eco*RI and cloned into the *Hin*dIII and *Eco*RI sites of pBluescript SK+. Second, a 0.2-kb PCR fragment containing the upstream end of *KanMX4* and the other set of linearization sites (fragment 2) digested with *Xho*I and *Hin*dIII was cloned into the *Xho*I and *Hin*dIII sites of the resulting plasmid. Third, a 1.1-kb PCR fragment containing the *URA3* marker (fragment 3) was digested with *Xho*I and *Hin*dIII and was cloned into the *Sal*I and *Hin*dIII sites of the resulting plasmid. Fourth, a 1.5-kb fragment containing a *GFP* reporter gene (fragment 4) was cut out from the plasmid pYOGM012 (Suzuki *et al.* 2011) using *Xho*I and *Hin*dIII and cloned into the *Sal*I and *Hin*dIII sites of the plasmid from the third round. Finally, a 2.3-kb PCR fragment containing sequences for overexpression (fragment 5) was digested using *Stu*I and cloned into blunt ends generated using Klenow from the sole *Hin*dIII site of the resulting plasmid. All PCR fragments used for cloning were prepared using Phusion polymerase following a protocol from the manufacturer (New England Biolabs). A clone containing fragment 5 in the desired orientation was identified using restriction analysis with *Xho*I. The correct orientation was confirmed with Sanger sequencing data. The sequence of the pYOGM081 plasmid has been deposited into GenBank (GenBank accession number: KJ094414).

To generate a plasmid carrying a gene insertion module that does not contain the HA-tag (pTJH001), pYOGM081 was amplified using the primers HAless-*Hin*dIII_F and HAless-*Hin*dIII_R (Table S1). The resulting fragment was digested using *Hin*dIII and self-ligated. To generate each of the modules (still containing the HA-tag) carrying the *ADH1* (pTJH002), *TEF* (pTJH003), and *CUP1* (pTJH004) promoters, the promoter was amplified using the Fwd and Rev primers for each promoter and the yeast BY4741 genomic DNA or pFA6a-kanMX4 (Wach *et al.* 1994) as a template. For each promoter, the pYOGM081 backbone was amplified using the corresponding pYOGM081_Fwd and pYOGM081_Rev primers. The promoter and the backbone were combined using Gibson assembly (Gibson *et al.* 2009). Cloning junctions were confirmed using Sanger sequencing.

### Generation of ProMonsters

Gateway entry clones containing human *PSTK*, *SPS1*, *SBP2*, *SBP2L*, *SECP43*, and *RPL30* were from the Human ORFeome Collection (Lamesch *et al.* 2007). The coding regions of human *SPS2*, *SECS*, *EEFSEC*, mouse *MsrA*, and *Caenorhabditis elegans SPS2* were amplified from cDNA clones (Open Biosystems), mouse liver cDNA or *C. elegans* total cDNA, respectively, and cloned into pDONR/Zeo vector (Life Technologies). Exogenous genes were transferred from the Gateway entry clones into the plasmids containing the iGM gene insertion cassettes using the Gateway LR reaction as described by the manufacturer.

*MAT***a** *KanMX4*-deletion strains were transformed with one of the 11 gene insertion cassettes constructed with the GFP insertion module, and transformants were selected via the *URA3* marker. Integration of the GFP insertion cassettes was confirmed using PCR with a primer complimentary to the 3′ end of the inserted human gene paired with a locus-specific primer from the 3′ flanking sequence of the deleted yeast open reading frame (ORF). This allowed for simultaneously confirming the targeting of the correct yeast locus and the presence of the exogenous gene in a single PCR reaction. We then individually mated each GFP-marked insertion strain with two MATα strains, one carrying *GMToolkit*-**a** and the other carrying *GMToolkit-α* (GMToolkit stands for “green monster toolkit”) constructs at the *CAN1* locus, respectively. *GMToolkit*-**a** and *GMToolkit-α* contain *rtTA* (the Tet activator necessary for the induction of GFP expression) (Gossen *et al.* 1995) and either *KanMX4* and *STE2pr-Sp-his5* (*GMToolkit*-**a**) or *NatMX4* and *STE3pr-LEU2* (*GMToolkit-α*) markers that facilitate sexual assortment of yeast strains (Suzuki *et al.* 2011). *KanMX4* and *NatMX4* markers can be used for selection of diploids carrying both *GMToolkit*-**a** and *GMToolkit-α*, whereas *Schizosaccharomyces pombe his5* under the control of the MAT**a**-specific *STE2* promoter and *LEU2* driven by the MATα-specific *STE3* promoter allow selection of haploid cells of the corresponding mating type. Mated diploids were selected for Ura^+^, as well as G418- and Nat-resistance conferred by *GMToolkit*-**a** and *GMToolkit-α*, respectively. The resulting strains were sporulated, and haploid MAT**a** and MATα ProMonster strains carrying both a *GFP* insertion and a *GMToolkit* were established by Ura^+^, as well as His^+^ and Leu^+^ selections, respectively. Expression of the exogenous genes introduced into the ProMonster strains was confirmed by Western blotting with HA-tag–specific antibodies.

### Flow cytometry enrichment and microscopy

The protocol for the enrichment of strains carrying multiple *GFP* copies using the Green Monster method has been published elsewhere (Suzuki *et al.* 2012). Flow cytometry enrichment was performed on a BD Aria sorter (BD Biosciences) equipped with a 488-nm (blue) laser for GFP (FL1) detection. For microscopy, exponentially growing cells were analyzed for GFP fluorescence with Leica TCS SP2 AOBS confocal laser scanning microscope. The ZEN 2009 Light Edition software was used to process the generated images.

### mRNA sequencing

Yeast cultures were grown to OD_600_ 0.5 in 50 ml of a medium containing 1.0% yeast extract, 2.0% peptone, 2.0% raffinose, and 0.1% glucose. Expression of proteins was induced by adding galactose to 2% into the cultures and incubating the cultures at 30° for an additional 60 min. Cells were collected by centrifugation, and the pellets were scraped with spatula, flash-frozen in liquid nitrogen, and stored at −80°. RNA was isolated using mRNA DIRECT kit (Life Technologies) according to manufacturer’s instructions. Construction of RNA-seq libraries was performed as previously described (Labunskyy *et al.* 2014), and the libraries were sequenced on the Illumina HiSeq2000 platform. RNA-seq reads were aligned to the *S. cerevisiae* genome from the Saccharomyces Genome Database (SGD; http://www.yeastgenome.org/, release number R64-1-1) and sequences of the introduced exogenous genes. Sequence alignment was performed using Bowtie software v.0.12.7 (Langmead *et al.* 2009) allowing two mismatches per read. To analyze expression of exogenous genes, rpkm (reads per kilobase per million mapped reads) values, which represent the number of reads normalized to gene length and total number of mapped reads, were calculated for each gene. An average rpkm value for two biological replicates is shown.

## Results

### Design of the iGM insertion module

Here, we describe the iGM set of expression vectors for rapid and facile introduction of multiple exogenous genes and assembly of the engineered loci into high-order multi-insertion strains in yeast (Figure 1A). A critical component of the iGM vectors is the universal gene insertion cassette. The insertion module is designed to introduce any exogenous gene along with the *GFP* quantitative marker into any locus of the yeast genome via homologous recombination (Figure 1B). Each expression cassette has a single *GFP* copy placed under the control of a promoter bearing two copies of the *tetO* regulatory unit (*tetO_2_pr*), a *URA3* transformation marker, and a Gateway cloning site preceded by either an inducible or a constitutive promoter and followed by a hemagglutinin (HA) epitope tag. For the construction of iGM expression cassettes, we chose two inducible promoters, *GAL1-10* and *CUP1*, that allow for inducible expression of exogenous genes by switching to galactose as a carbon source and by changing copper levels, respectively, in the culture media. In addition, we have developed alternative expression cassettes containing two moderate-level constitutive promoters, *ADH1* and *TEF*, and an additional version of the insertion module that contains the *GAL1-10* promoter but lacks the HA-tag. Exogenous genes of interest can be efficiently amplified and cloned into any of the insertion modules using the Gateway cloning system (Walhout *et al.* 2000), which is based on bacteriophage lambda site-specific recombination (Landy 1989). The insertion module is flanked by sequences with homology to *KanMX4*, as well as unique restriction sites. On introduction of an exogenous gene into the Gateway vector and excision of the insertion module, the released fragment can be targeted into any locus in the yeast genome, for which there is an available strain from the *KanMX4* yeast knockout collection. Integration of the insertion module into the genome is achieved by homologous recombination between the insertion module and a *KanMX4* deletion cassette in the knockout strain. A “neutral” locus, where the deletion of the gene does not result in any growth defect or synthetic lethal interaction with other deletion loci, is often preferable for the purpose of inserting exogenous genes.

Once each of the exogenous genes is integrated in an individual strain termed a ProMonster strain, the multiple gene insertions are combined into a single strain by virtue of repeated cycles of yeast mating, sporulation, and flow-cytometric selection of the Green Monster method (Figure 1C). This sexual cycling process is facilitated by haploid- and diploid-selection markers present in the *GMToolkit-***a** and *GMToolkit-α* constructs integrated into these strains (Suzuki *et al.* 2011).

### Testing iGM components

**Figure 2:**
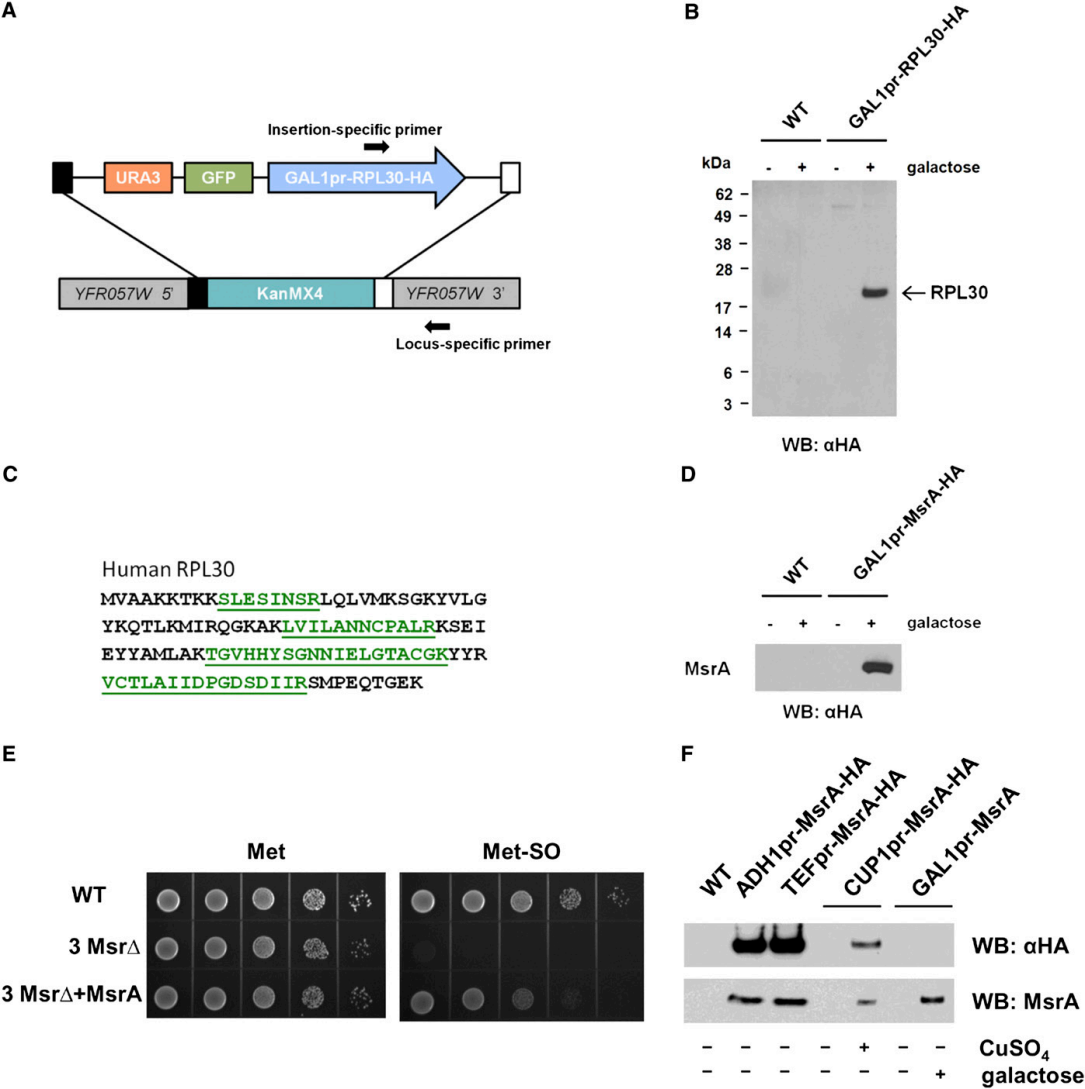
Testing the iGM insertion module. (A) Yeast *YFR057W* ORF is replaced by an insertion module containing human *RPL30* gene that was cloned under control of *GAL1-10* promoter and linked to an inducible *GFP* and the *URA3* marker. Positions of the primers for genotyping are indicated by arrows. (B) Expression of the human *RPL30* gene in a strain containing the *RPL30* insertion module was detected by Western blotting with HA-tag–specific antibodies. Expression of the protein was induced by galactose. (C) Peptide sequences identified in a yeast strain containing the *RPL30* insertion module by LC-MS/MS. RPL30 protein was immunoprecipitated with HA-tag antibody, resolved by SDS-PAGE, and subjected to in-gel trypsin digestion. The peptides detected by mass spectrometry are shown in green. (D) Expression of the mouse MsrA protein in the 3 Msr∆ strain lacking all three Msr enzymes (*i.e.*, MsrA, MsrB, and fRMsr). The insertion module containing mouse *MsrA* gene under control of *GAL1-10* promoter was integrated into the 3 Msr∆ strain, and expression of the protein following galactose induction was detected by Western blotting with αHA antibodies. (E) Expression of the mouse MsrA rescued the growth of 3 Msr∆ strain missing all three yeast Msrs on medium containing a source of Met-SO. Yeast strains were analyzed for growth on Met-free SC medium supplemented with 20 mg/liter Met (left) or Met-SO (right). Cells were initially grown on SC liquid medium until OD_600_ reached 0.6, harvested, washed, diluted to OD_600_ of 0.1 in water, and serially spotted on agar SC plates containing galactose and respective Met or Met-SO sources. The plates were incubated at 30°, and images were taken 48 hr after plating. (F) Expression of the mouse MsrA protein using iGM vectors containing alternative promoters. Insertion modules containing the mouse *MsrA* gene under control of *ADH1*, *TEF*, and *CUP1* promoters or a version of the plasmid containing the *GAL1-10* promoter that lacks the HA-tag were integrated into *YER042W* locus, and expression of the protein was detected by Western blotting with either HA-tag or MsrA-specific antibodies. Where indicated, logarithmically growing cultures were induced to produce the protein by addition of 100 µM CuSO_4_ or 2% galactose for 4 hr.

To see if the GFP gene insertion module can be targeted into a specific locus in yeast and induced to express an exogenous gene, we constructed a Gateway entry clone containing the ORF of the human ribosomal protein L30 (RPL30) and transferred the *RPL30* gene to the insertion module containing the *GAL1-10* promoter using the Gateway LR reaction. The resulting insertion module was released from the plasmid and used for transformation of the yeast *yfr057wΔ* strain from the *KanMX4* deletion mutant library. Successful integration of the exogenous gene into the specific yeast locus was confirmed by PCR with a forward primer that anneals to the 3′ end of the human *RPL30* gene and a reverse primer that anneals to a locus-specific sequence in the 3′ flanking region of the deleted yeast ORF (Figure 2A). This allowed us to simultaneously confirm the correct placement of the module and the presence of the exogenous gene in a single PCR reaction. The *GAL1-10* inducible promoter is commonly used for expression of exogenous genes in yeast (Romanos *et al.* 1992). The human RPL30 protein was produced in the yeast strain carrying the insertion module on culture of cells in the presence of galactose, as assayed using Western blotting with HA-tag–specific antibodies, whereas basal expression was undetectable in the uninduced cells (Figure 2B). In addition, the C-terminal HA-epitope tag was used to purify the human RPL30 protein by immunoprecipitation, and the production of the full-length protein was confirmed using LC-MS/MS (Figure 2C).

To demonstrate that iGM tools can be used to express a functional protein, we incorporated a gene encoding mouse methionine-*S*-sulfoxide reductase (MsrA) into the insertion module. Methionine sulfoxide reductases (Msrs) are widespread antioxidant enzymes that catalyze the reduction of methionine sulfoxide (Met-SO) back to methionine (Met) in a stereospecific manner (Stadtman *et al.* 2002). Because yeast encodes three Msr proteins in its genome, we integrated the insertion module containing mouse *MsrA* into a yeast mutant, in which all three endogenous Msr genes were deleted (3 Msr∆) (Le *et al.* 2009), and tested the ability of the exogenously introduced MsrA to complement the function of its yeast homolog (Figure 2, D and E). We found that expression of mouse MsrA protein on galactose induction supported the growth of the 3 Msr∆ mutant on media lacking Met and supplemented with Met-SO. Therefore, the functional mouse MsrA was responsible for reduction of Met-SO that could be used as a source of Met in these cells, because the triple Msr mutant was only able to grow on media supplemented with Met but not on Met-SO–containing media. In addition, we confirmed the utility of the alternative expression cassettes each containing the *ADH1*, *TEF*, or *CUP1* promoter and the version of the vector containing the *GAL1-10* promoter that lacks the HA-tag by driving the expression of the mouse MsrA protein from each of the constructs in yeast and detecting the expression using Western blotting with antibodies specific to either HA-tag or MsrA (Figure 2F).

### Expression of human Sec biosynthesis genes in yeast

**Figure 3:**
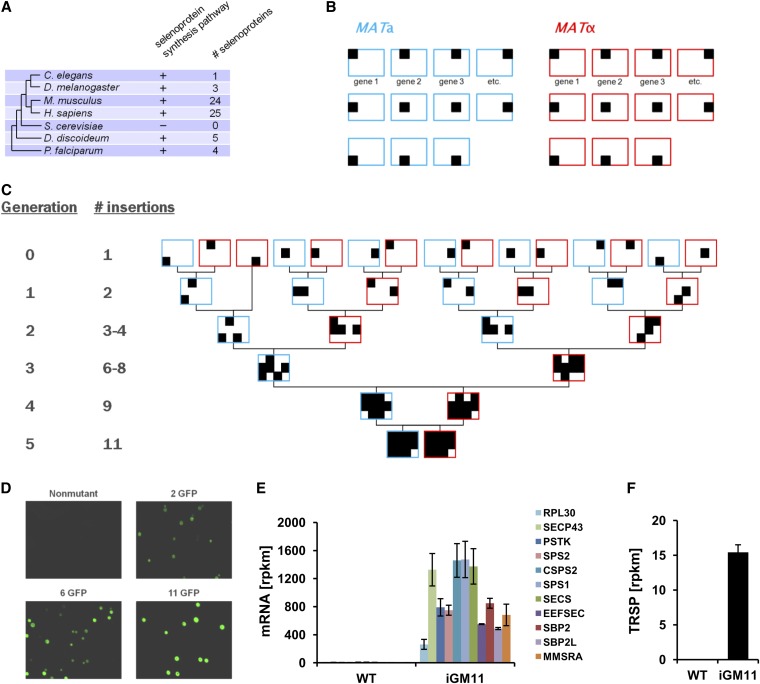
Generation of the 11-insertion Green Monster strains (iGM11). (A) A diagram showing phylogenetic tree and conservation of the selenoprotein synthesis pathway in different clades. The presence of the selenoprotein synthesis machinery and number of selenoproteins encoded in the genome are indicated. (B) Individual GFP insertion strains of two different mating types were generated for the 11 human genes involved in selenoprotein synthesis. Blue rectangles represent MAT**a** strains and red rectangles represent MATα strains. Specific positions of filled squares (▪) in each rectangle indicate inserted exogenous genes. (C) To generate yeast strains with multiple insertions of exogenous genes, individual strains carrying a single engineered locus linked to an inducible *GFP* marker gene were subjected to repeated cycles of the Green Monster process. The number of sexual cycling rounds and the number of obtained insertions in strains are shown on the left. (D) Representative images of a nonmutant strain and strains containing 2, 6, and 11 GFP insertion modules. Identical exposure, brightness, and contrast settings were used for fluorescence imaging. (E) Quantification of mRNA expression levels in the iGM11 mutant using RNA-seq. To analyze expression of exogenous genes, mRNA reads obtained by sequencing the transcriptome of the iGM11 mutant were aligned to the sequences of the introduced genes. The rpkm (reads per kilobase per million mapped reads) values represent the number of reads normalized to gene length and total number of mapped reads. Error bars indicate SEM. Measurements from biological replicates are shown. (F) RNA-seq read density (rpkm) aligned to the sequence of *TRSP* gene encoding human Sec tRNA. Error bars indicate SEM. Measurements from biological replicates are shown.

To test if multiple gene insertions can be assembled into high-order multi-insertion strains, we applied the iGM strategy to insert genes encoding components of the human pathway for selenoprotein synthesis, which is absent in the yeast genome. Sec is the twenty-first amino acid in the genetic code, which is encoded by the UGA codon (Hatfield and Gladyshev 2002). UGA has a dual role, as it serves both to direct the incorporation of Sec and to terminate peptide chain elongation depending on the presence of several *cis*- and *trans*-acting factors. Organisms that utilize Sec have evolved a complex protein network required for its biosynthesis and incorporation into proteins. There are 25 genes encoding selenoproteins in humans (Kryukov *et al.* 2003); however, budding yeast completely lacks selenoproteins and components of the Sec biosynthesis and insertion pathways (Figure 3A). Because the fungal branch is one of the most proximal branches to human that lack the Sec pathway, expression of human Sec machinery in yeast would provide an ideal test-bed for the functional characterization of individual factors that influence Sec incorporation.

Human Sec tRNA gene (*TRSP*) and the following 10 genes that are known to function in eukaryotic Sec biosynthesis and incorporation (Squires and Berry 2008; Xu *et al.* 2007) were separately introduced into the insertion module using the Gateway cloning system: human *PSTK*; *SECS*; *SPS1*; *SPS2*; *SBP2*; *SBP2L*; *EEFSEC*; *SECP43*; *RPL30*; and *C. elegans SPS2* (*cSPS2*). Each of the 10 genes was cloned into the insertion module adjacent to an inducible *GAL1-10* promoter (Romanos *et al.* 1992). For expression of human Sec tRNA, the *TRSP* gene was cloned into the insertion module lacking the *GAL1-10* promoter and placed under the control of the promoter of a gene for an Arg-tRNA from *S. cerevisiae* (Francis and Rajbhandary 1990). Using homologous recombination, the gene insertion cassettes were then integrated into candidate neutral loci in the yeast genome, which were selected based on functional predictions from co-fitness and synthetic lethal interaction data (Costanzo *et al.* 2010; Hillenmeyer *et al.* 2008; Tong *et al.* 2004). In addition, we chose to replace *YER042W*, *YCL033C*, and *YKL069W* ORFs, which encode components of the pathway for methionine sulfoxide reduction in yeast (Le *et al.* 2009), with three genes (see below) of the human Sec biosynthesis pathway.

### Generating Green Monster strains expressing multiple inserted genes

Next, we used the Green Monster strategy to assemble 11 GFP-marked insertion loci into a single strain. To facilitate sexual cycling, we introduced the *GMToolkit-***a** and *GMToolkit-α* constructs into the individual insertion strains via yeast mating and generated 22 ProMonster strains (Figure 3B) of two different mating types (11 MAT**a** and 11 MATα strains). We then used pairwise mating between MAT**a** and MATα strains to generate diploids and sporulated the resulting diploid strains. After sexual recombination, a mixture of haploid cells containing a different number of insertions (0, 1, or 2) was subjected to enrichment using flow cytometry to select cells with the highest GFP fluorescence intensity. After the first round of the Green Monster process, 96% of the genotyped haploids were double insertion strains (n = 77) as compared to 24% (predicted recombination rate of two unlinked genes is 25%) for the unsorted haploids (n = 83), when strains from all crosses except one were combined. However, in one of the crosses, two insertions were located on the same chromosome, ∼27 kb apart. For this cross with closely linked insertions, 25% (n = 4) and 0% (n = 6) of the genotyped haploids were double insertion strains for the sorted and unsorted samples, respectively. The genotyped double insertion mutant strains were then individually mated with double mutants of the opposite mating type and the cycle was repeated until all of the 11 gene insertions were combined in the single strain iGM11 (Figure 3C). We were able to generate the iGM11 monster in five rounds of the Green Monster process and genotyping. Each round was completed in 13 days, with the total time of 65 days spent for constructing the iGM11 monsters. Analysis of the iGM strains using microscopy demonstrated a correlation of GFP fluorescence intensity with the number of insertions (Figure 3D). The integrity of the generated iGM11 strain was verified by sequencing the whole genome (Figure S2 and Figure S3), and expression of all 11 exogenous genes that were introduced into the iGM11 strain was confirmed both at the level of mRNA (Figure 3, E and F) and protein (Figure S4) using RNA-seq and Western blotting with HA-tag–specific antibodies, respectively.

### Strategy for identification of factors that influence Sec incorporation

**Figure 4:**
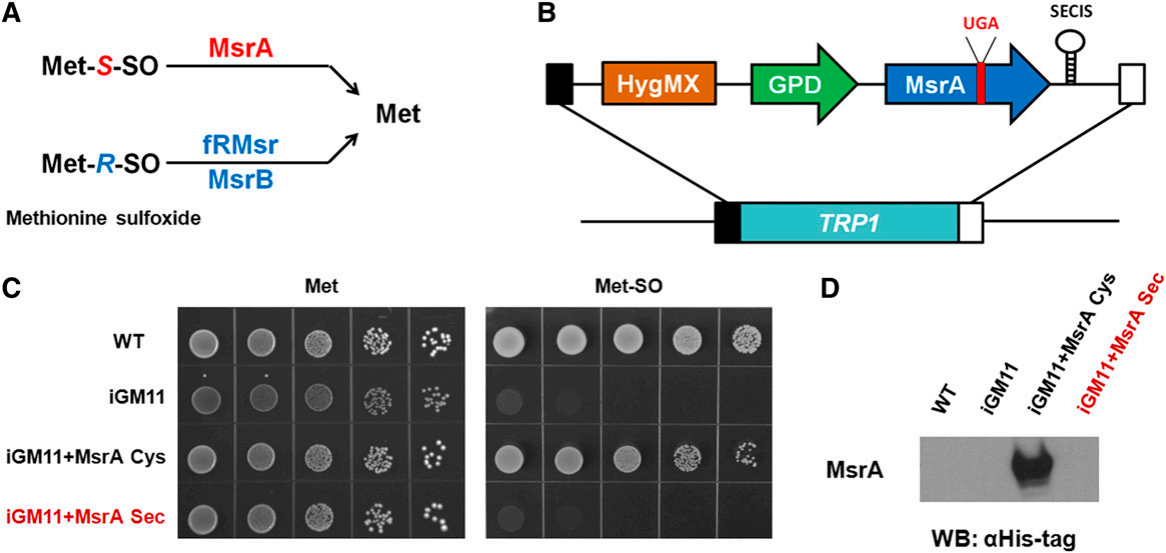
Strategy for identification of factors that influence Sec incorporation using MsrA selenoprotein reporter. (A) Three yeast Msr genes that catalyze the reduction of Met-SO were deleted in iGM11 strain to allow conditional selection of cells expressing functional MsrA selenoprotein. *S*-(Met-*S*-SO) and *R*-diastereomers (Met-*R*-SO) of methionine sulfoxide are reduced back to Met by MsrA and MsrB/fRMsr methionine sulfoxide reductases, respectively. (B) *GPDpr-MsrA(C72U)-SECIS-HphMX4* reporter was integrated into the *TRP1* locus of the iGM11 strain. The reporter contains mouse *MsrA* gene cloned under the control of the *GPD* promoter and a modified SECIS element from *Toxoplasma gondii* selenoprotein T (Novoselov *et al.* 2007). We introduced a C-terminal His-tag into the mouse *MsrA* sequence and replaced active site Cys72 residue with Sec. UGA codon corresponding to Sec is shown in red. (C) Mouse *MsrA* gene reporters possessing redox-active Cys (MsrA-Cys) or Sec (MsrA-Sec) were each separately integrated into iGM11 strain. Yeast strains were analyzed for growth on Met-free SC medium supplemented with 20 mg/liter Met (left) or Met-SO (right). Cells were initially grown on SC liquid medium until OD_600_ reached 0.6, harvested, washed, diluted to OD_600_ of 0.1 in water, and serially spotted on agar SC plates containing galactose and respective Met or Met-SO sources. The plates were incubated at 30°, and images were taken 48 hr after plating. (D) Expression of the reporters in the iGM11 mutant was detected by Western blotting with His-tag–specific antibodies.

To facilitate the characterization of the selenoprotein synthesis pathway and to enable the identification of factors required for Sec incorporation, we developed a reporter construct that could encode a selenoprotein form of mouse MsrA (Kim *et al.* 2006). In yeast, the methionine sulfoxide reduction pathway consists of three enzymes (MsrA, MsrB, and fRMsr) that catalyze the repair of oxidized Met and Met residues in proteins in a stereospecific manner (Figure 4A). MsrA exhibits specificity for methionine-*S*-sulfoxide (Met-*S*-SO), whereas MsrB and fRMsr are specific for the reduction of the second stereoisomer, methionine-*R*-sulfoxide (Met-*R*-SO). These enzymes have been shown to support the growth of yeast cells on Met-SO, which consists of a racemic mixture of two stereoisomers (Le *et al.* 2009). Therefore, the growth of cells in the presence of Met-SO as a source of Met can be used as a proxy for synthesis and incorporation of Sec, when a condition is generated where only a selenoprotein provides the Msr activity. The *GPDpr-MsrA(C72U)-SECIS-HphMX4* reporter (MsrA-Sec) was integrated into the endogenous *TRP1* locus of the iGM11 strain (Figure 4B). To inactivate the endogenous Met-SO reduction system in the final strain, we used deletion alleles for *YER042W*, *YCL033C*, and *YKL069W* encoding yeast MsrA, MsrB, and fRMsr, respectively, when we introduced components of the human Sec pathways to generate iGM11. We observed that the growth of this strain was significantly decreased in the medium where Met was replaced with Met-SO (Figure 4C). Because the selenoprotein form of mouse MsrA contains a UGA codon and a Sec insertion sequence (SECIS) element in its 3′-end untranslated (3′-UTR) region, which are each necessary to decode Sec, the functional MsrA-Sec reporter will be only expressed when Sec is co-translationally inserted into the protein, thus allowing cells to utilize methionine sulfoxide as a source of methionine. We also introduced a C-terminal His-tag into the MsrA reporter gene (Figure 4D), which together with Met-SO selection strategy outlined above, can be used for detection of MsrA-Sec and identification of factors that influence Sec incorporation. Although the iGM11 strain containing 11 of the selenoprotein synthesis machinery components did not support the expression of the MsrA-Sec reporter, we found that a cysteine-containing form of the reporter (MsrA-Cys) similarly introduced into iGM11 mutant rescued the growth of cells in the presence of Met-SO, suggesting that MsrA is efficiently expressed and results in production of a functional enzyme. Therefore, the Met-SO selection strategy can be used as an effective screening tool for identification of additional requirements for selenocysteine synthesis and incorporation.

## Discussion

We developed the iGM strategy for routinely engineering *S. cerevisiae* strains carrying multiple gene insertions, which can be used for functional studies of complex multi-gene pathways. As a proof of principle, we applied this approach to constructing a yeast system for studying the human Sec biosynthesis and insertion pathway and identifying requirements for selenoprotein synthesis. Expression of selenoproteins requires a set of specific factors that decode an in-frame UGA codon as Sec instead of translation termination. These factors include a *cis*-acting mRNA sequence (SECIS) and three *trans*-acting factors: Sec tRNA; Sec-specific elongation factor (EEFSEC); and SECIS binding protein 2 (SBP2) (Driscoll and Copeland 2003). Moreover, unlike other amino acids, Sec is synthesized on its own tRNA in a process catalyzed by phosphoseryl-tRNA kinase (PSTK), selenophosphate synthetase 2 (SPS2), and selenocysteine tRNA synthase (SECS) (Xu *et al.* 2007). Although the functions of core elements of the selenoprotein synthesis machinery are well-studied, the mechanism by which Sec is incorporated into proteins in eukaryotes is not completely understood, and it is not known whether other *trans*-acting factors are required. To develop an expression system using yeast cells for characterization of the human pathway for selenoprotein biosynthesis, we introduced six genes encoding Sec tRNA, PSTK, SPS2, SECS, SBP2, and EEFSEC into *S. cerevisiae* using the iGM strategy. In addition, we incorporated four factors that have been implicated in Sec incorporation, but whose function is not known: SECP43 (a protein that was shown to bind to Sec tRNA); SPS1 (a homolog of selenophosphate synthetase 2); and SBP2L and RPL30 (two proteins that are known to bind SECIS element). Because expression of human SPS2 would require functionally active Sec biosynthesis machinery (human SPS2 is a selenoprotein), we also expressed a cysteine-containing form of SPS2 from *C. elegans* to complement its function. Altogether, we generated 22 strains (11 strains of each of the two mating types) that encode individual components of the human pathway for selenoprotein biosynthesis and we also produced a multi-iGM strain (iGM11), in which all of the 11 components were integrated into the yeast genome. These strains provide tools for functional characterization of the individual protein factors involved in selenoprotein synthesis and studying the mechanism of mammalian Sec biosynthesis and insertion by exploiting a cellular environment of a simple eukaryote, *S. cerevisiae*. In addition, using the iGM method, a number of intermediate strains containing different numbers of insertions were developed. Because the introduced genes stay modular, additional crosses can be used to generate genotypes varying in the combinations of exogenous genes present. Therefore, these strains are expected to be useful for examining sequential steps of Sec biosynthesis and insertion pathway in a stepwise fashion.

It should be acknowledged that other *trans*-acting factors may be required for Sec insertion. To facilitate discovery of previously unidentified essential factors that are required for Sec incorporation in humans, we developed a screening procedure using a selenoprotein reporter construct encoding methionine sulfoxide reductase MsrA. This protein contains TGA codon within the ORF and a SECIS element in the 3′-end untranslated region. When TGA (UGA in messenger RNA) is translated as Sec, a functional MsrA protein will be expressed, thus allowing cells to utilize methionine sulfoxide as a source of methionine. This approach allows for conditional selection and can be used for screening human cDNA libraries to identify factors that are required for Sec incorporation.

Selenoproteins are present in all three lines of descent, eukaryota, archaea, and eubacteria, but were lost in some species, including fungi. A recent study using *in vitro* assays has shown that ribosomes from species that do not utilize Sec might be intrinsically incompatible with Sec incorporation (Gupta *et al.* 2013). This observation suggests that additional humanization of the translation machinery or the cellular environment that surrounds the introduced human selenoprotein synthesis pathway may be required to support efficient Sec incorporation in yeast. Our system provides tools for replacement of yeast ribosome components with human counterparts, therefore offering means to identify potential ribosome-specific factors that govern Sec insertion instead of translation termination.

Studying the mechanism of mammalian selenoprotein synthesis and reconstruction of Sec insertion machinery is a focus of current research in the field of selenium biology. The iGM strains developed here are expected to facilitate these studies and can be used as a resource for detailed characterization of the mechanism of Sec insertion and to assign functions to currently uncharacterized *trans*-acting factors that influence Sec incorporation.

## Supplementary Material

Supporting Information
