# Supplementary material for: The Insertion Green Monster (iGM) Method for Expression of Multiple Exogenous Genes in Yeast
Source: G3 (Bethesda). 2014 Apr 28;4(7):1183–91. doi: 10.1534/g3.114.010868 (PMC4455768; doi:10.1534/g3.114.010868)
Supplement: Supporting Information [file supp_4_7_1183__index.html]

The Insertion Green Monster (iGM) Method for Expression of Multiple Exogenous Genes in Yeast — Supporting Information 

# The Insertion Green Monster (iGM) Method for Expression of Multiple Exogenous Genes in Yeast

## Supporting Information for Labunskyy *et al.*, 2014

**Files in this Data Supplement:**

- Supporting Information - Figures S1-S4 and Tables S1-S2 (PDF, 2 MB)
- Figure S1 - Sequence of the pYOGM081 plasmid containing the iGM gene insertion module. (PDF, 2 MB)
- Figure S2 - Integration of the metazoan Sec biosynthesis and insertion genes into the yeast genome. (PDF, 464 KB)
- Figure S3 - Sequencing of the whole genome confirms deletion of the yeast genes. (PDF, 450 KB)
- Figure S4 - Expression analysis of the exogenous genes introduced into the yeast genome using iGM method. (PDF, 349 KB)
- Table S1 - PCR primers used for generating a universal gene insertion module. (PDF, 132 KB)
- Table S2 - List of genotyping primers used in this study. (PDF, 116 KB)
