# Supplementary material for: The Insertion Green Monster (iGM) Method for Expression of Multiple Exogenous Genes in Yeast
Source: G3 (Bethesda). 2014 Apr 28;4(7):1183–91. doi: 10.1534/g3.114.010868 (PMC4455768; doi:10.1534/g3.114.010868)
Supplement: Supporting Information [file supp_g3.114.010868_010868SI.pdf]

G3: Genes | Genomes | Genetics

The Insertion Green Monster (iGM) Method for Expression of Multiple Exogenous Genes in Yeast

Vyacheslav M. Labunskyy,*[,1] Yo Suzuki,[†,1] Timothy J. Hanly,[†] Ayako Murao,[†] Frederick P. Roth,[‡,§,2] and Vadim N. Gladyshev*[,2]

*Division of Genetics, Department of Medicine, Brigham and Women's Hospital and Harvard Medical School, Boston, Massachusetts 02115, [†]Department of Synthetic Biology and Bioenergy, J. Craig Venter Institute, La Jolla, California 92037, [‡]Donnelly Centre and Departments of Molecular Genetics and Computer Science, University of Toronto, Toronto, Ontario M5S 3E1, Canada, and [§]Lunenfeld-Tanenbaum Research Institute, Mt. Sinai Hospital, Toronto, Ontario M5T 3L9, Canada

[1]These authors contributed equally to this work.

[2]Corresponding authors: University of Toronto, Donnelly Centre, 160 College Street, Toronto, Ontario M5S 3E1, Canada. E-mail: fritz.roth@utoronto.ca; Division of Genetics, Department of Medicine, Brigham and Women's Hospital, 77 Avenue Louis Pasteur, Boston, MA 02115. E-mail: vgladyshev@rics.bwh.harvard.edu

DOI: 10.1534/g3.114.010868

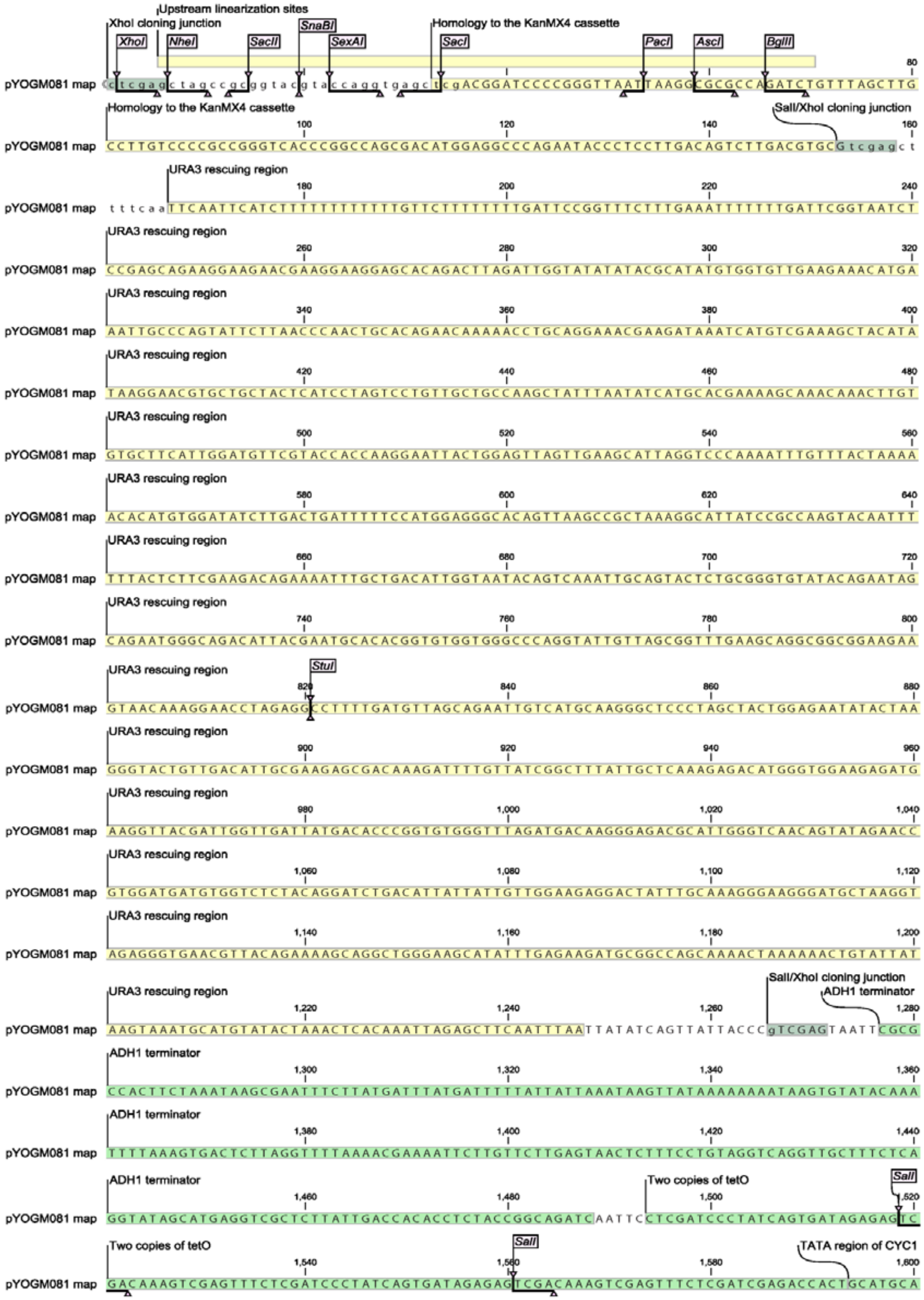

Figure S1 Sequence of the pYOGM081 plasmid containing the iGM gene insertion module. In a version of the plasmid not containing the HA-tag (pTJH001), nucleotides 4,954–5,101 are removed and replaced with a stop codon (TAA) and a *Hin*dIII site (Materials and Methods). In plasmids containing the *ADH1* (pTJH002), *TEF* (pTJH003), and *CUP1* (pTJH004) promoters for driving the expression of a gene introduced between the Gateway cloning sites, nucleotides 2,755–3,232 are replaced with the respective sequences shown below (Materials and Methods).

Figure S1 (continued).

TATA region of CYC1
1,620 1,640 1,660 1,680
pYOGM081 map TGTGCTCTGTATGTATATAAAACTCTTGTTTTCTTCTTTTCTCTAAATATTCTTTCCTTATACATTAGGTCCTTTGTAGC

TATA region of CYC1 GFP coding sequence
1,700 1,720 1,740 1,760
pYOGM081 map ATAAATTACTATACTTCTATAGACACGCAAACACAAATACACACACTAAATTACCGGATCAATTCGGGGGATCCATGAGT

GFP coding sequence
1,780 1,800 1,820 1,840
pYOGM081 map AAAGGAGAAGAACTTTTCACTGGAGTGGTCCCAGTTCTTGTTGAATTAGATGGCGATGTTAATGGGCAAAAATTCTCTGT

GFP coding sequence
1,860 1,880 1,900 1,920
pYOGM081 map CAGTGGAGAGGGTGAAGGTGATGCAACATACGGAAAACTTACCCTTAATTTTATTTGCACTACTGGGAAGCTACCTGTTC

GFP coding sequence
1,940 1,960 1,980 2,000
pYOGM081 map CATGGCCAACACTTGTCACTACTTTCTCTTATGGTGTTCAATGCTTCTCAAGATACCCAGATCATATGAAACAGCATGAC

GFP coding sequence
2,020 2,040 2,060 2,080
pYOGM081 map TTTTTCAAGAGTGCCATGCCCGAAGGTTATGTACAGGAAAGAACTATATTTTACAAAGATGACGGGAACTACAAGACACG

GFP coding sequence
2,100 2,120 2,140 2,160
pYOGM081 map TGCTGAAGTCAAGTTTGAAGGTGATACCCTTGTTAATAGAATCGAGTTAAAAGGTATTGATTTTAAAGAAGATGGAAACA

GFP coding sequence
2,180 2,200 2,220 2,240
pYOGM081 map TTCTTGGACACAAAATGGAATACAACTATAACTCACATAATGTATACATCATGGGAGACAAACCAAAGAATGGCATCAAA

GFP coding sequence
2,260 2,280 2,300 2,320
pYOGM081 map GTTAACTTCAAAATTAGACACAACATTAAAGATGGAAGCGTTCAATTAGCAGACCATTATCAACAAAATACTCCAATTGG

GFP coding sequence
2,340 2,360 2,380 2,400
pYOGM081 map CGATGGCCCTGTCCTTTTACCAGACAACCATTACCTGTCCACACAATCTGCCCTTTCCAAAGATCCCAACGAAAAGAGAG

GFP coding sequence
2,420 2,440 2,460 2,480
pYOGM081 map ATCACATGATCCTTCTTGAGTTTGTAACAGCTGCTAGGATTACACATGGCATGGATGAACTATACAAAtaaGGGCCCTGC

CYC1 terminator
2,500 2,520 2,540 2,560
pYOGM081 map AGGAGGGCCGCATCATGTAATTAGTTATGTCACGCTTACATTCACGCCCTCCCCCCACATCCGCTCTAACCGAAAAGGAA

CYC1 terminator
2,580 2,600 2,620 2,640
pYOGM081 map GGAGTTAGACAACCTGAAGTCTAGGTCCCTATTTATTTTTTTATAGTTATGTTAGTATTAAGAACGTTATTTATATTTCA

CYC1 terminator
2,660 2,680 2,700 2,720
pYOGM081 map AATTTTTCTTTTTTTTCTGTACAGACGCGTGTACGCATGTAACATTATACTGAAAACCTTGCTTGAGAAGGTTTTGGGAC

HindIII/StuI cloning junction
CYC1 terminator GAL1-10 promoter
2,740 2,760 2,780 2,800
pYOGM081 map GCTCGAAGGCTTTAATTTGCGGCCAAGCTcctGTACGGATTAGAAGCCGCCGAGCGGGCGACAGCCCTCCGACGGAAGAC

GAL1-10 promoter
2,820 2,840 2,860 2,880
pYOGM081 map TCTCCTCCGTGCGTCCTCGTCTTCACCGGTCGCGTTCCTGAAACGCAGATGTGCCTCGCGCCGCACTGCTCCGAACAATA

GAL1-10 promoter
2,900 2,920 2,940 2,960
pYOGM081 map AAGATTCTACAATACTAGCTTTTATGGTTATGAAGAGGAAAAATTGGCAGTAACCTGGCCCCACAAACCTTCAAATTAAC

GAL1-10 promoter
2,980 3,000 3,020 3,040
pYOGM081 map GAATCAAATTAACAACCATAGGATGATAATGCGATTAGTTTTTTAGCCTTATTTCTGGGGTAATTAATCAGCGAAGCGAT

GAL1-10 promoter
3,060 3,080 3,100 3,120
pYOGM081 map GATTTTTGATCTATTAACAGATATATAAATGGAAAAGCTGCATAACCACTTTAACTAATACTTTCAACATTTTCAGTTTG

GAL1-10 promoter
3,140 3,160 3,180 3,200
pYOGM081 map TATTACTTCTTATTCAAATGTCATAAAAGTATCAACAAAAAATTGTTAATATACCTCTATACTTTAACGTCAAGGAGAAA

Figure S1 (continued).

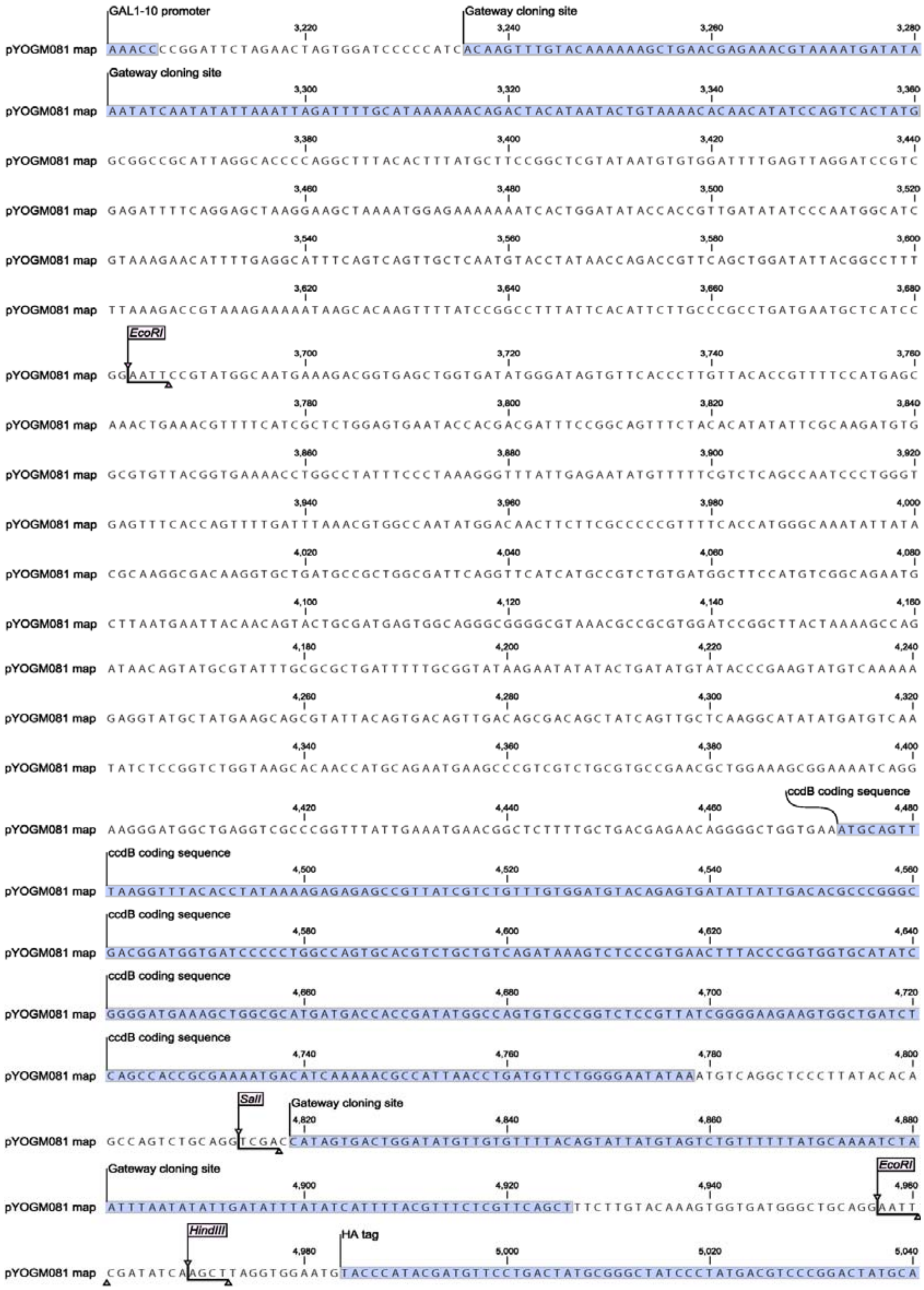

Figure S1 (continued).

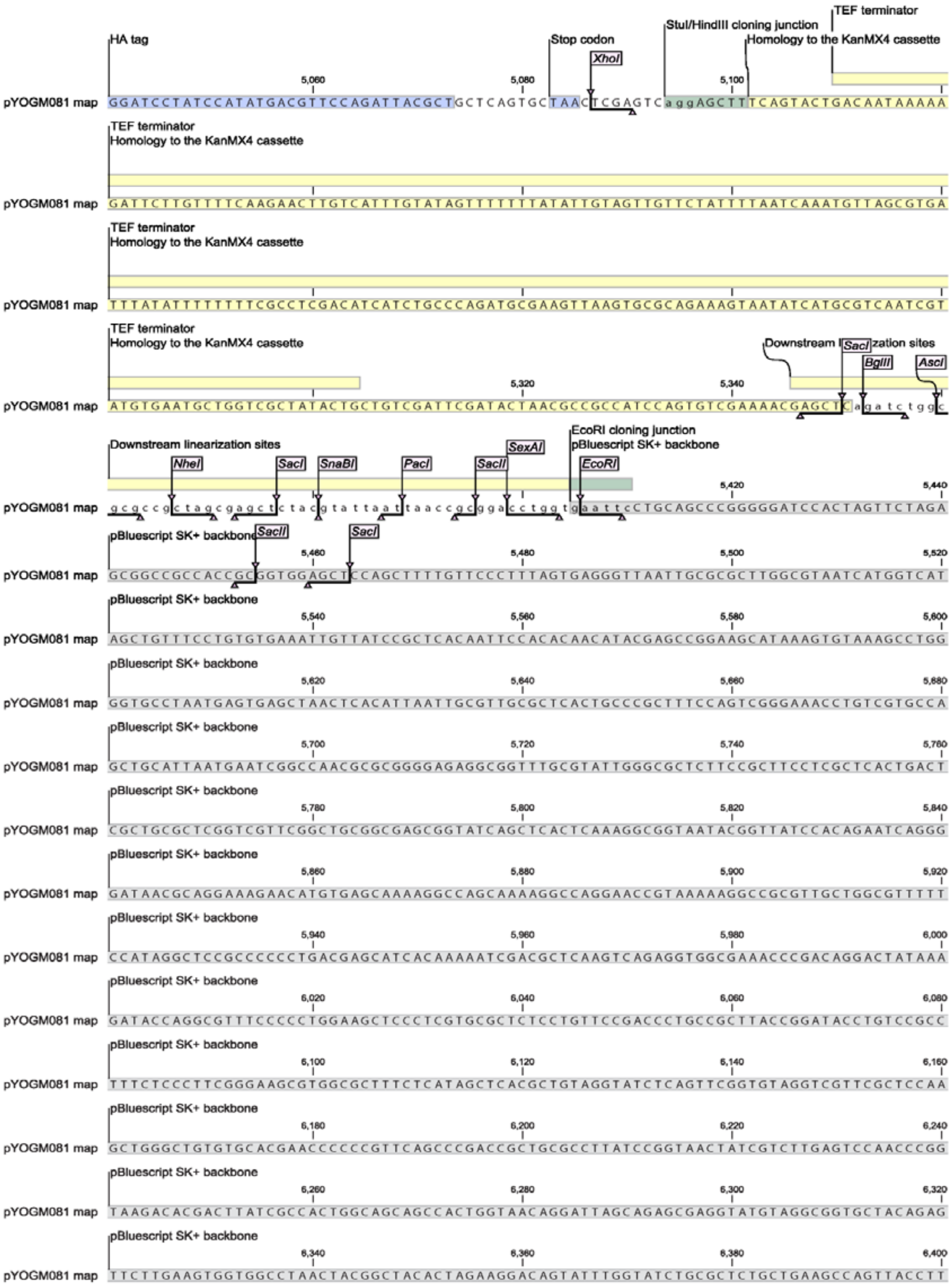

Figure S1 (continued).

```
pBluescript SK+ backbone
6,420 6,440 6,460 6,480
pYOGM081 map CGGAAAAAGAGTTGGTAGCTCTTGATCCGGCAAACAAACCACCGCTGGTAGCGGTGGTTTTTTTGTTTGCAAGCAGCAGA
pBluescript SK+ backbone
6,500 6,520 6,540 6,560
pYOGM081 map TTACGCGCAGAAAAAAAGGATCTCAAGAAGATCCTTTGATCTTTTCTACGGGGTCTGACGCTCAGTGGAACGAAAACTCA
pBluescript SK+ backbone
6,580 6,600 6,620 6,640
pYOGM081 map CGTTAAGGGATTTTGGTCATGAGATTATCAAAAAGGATCTTCACCTAGATCCTTTTAAATTAAAAATGAAGTTTTAAATC
pBluescript SK+ backbone
6,660 6,680 6,700 6,720
pYOGM081 map AATCTAAAGTATATATGAGTAAACTTGGTCTGACAGTTACCAATGCTTAATCAGTGAGGCACCTATCTCAGCGATCTGTC
pBluescript SK+ backbone
6,740 6,760 6,780 6,800
pYOGM081 map TATTTCGTTCATCCATAGTTGCCTGACTCCCCGTCGTGTAGATAACTACGATACGGGAGGGCTTACCATCTGGCCCCAGT
pBluescript SK+ backbone
6,820 6,840 6,860 6,880
pYOGM081 map GCTGCAATGATACCGCGAGACCCACGCTCACCGGCTCCAGATTTATCAGCAATAAACCAGCCAGCCGGAAGGGCCGAGCG
pBluescript SK+ backbone
6,900 6,920 6,940 6,960
pYOGM081 map CAGAAGTGGTCCTGCAACTTTATCCGCCTCCATCCAGTCTATTAATTGTTGCCGGGAAGCTAGAGTAAGTAGTTCGCCAG
pBluescript SK+ backbone
6,980 7,000 7,020 7,040
pYOGM081 map TTAATAGTTTGCGCAACGTTGTTGCCATTGCTACAGGCATCGTGGTGTCACGCTCGTCGTTTGGTATGGCTTCATTCAGC
pBluescript SK+ backbone
7,060 7,080 7,100 7,120
pYOGM081 map TCCGGTTCCCAACGATCAAGGCGAGTTACATGATCCCCCATGTTGTGCAAAAAAGCGGTTAGCTCCTTCGGTCCTCCGAT
pBluescript SK+ backbone
7,140 7,160 7,180 7,200
pYOGM081 map CGTTGTCAGAAGTAAGTTGGCCGCAGTGTTATCACTCATGGTTATGGCAGCACTGCATAATTCTCTTACTGTCATGCCAT
pBluescript SK+ backbone
7,220 7,240 7,260 7,280
pYOGM081 map CCGTAAGATGCTTTTCTGTGACTGGTGAGTACTCAACCAAGTCATTCTGAGAATAGTGTATGCGGCGACCGAGTTGCTCT
pBluescript SK+ backbone
7,300 7,320 7,340 7,360
pYOGM081 map TGCCCGGCGTCAATACGGGATAATACCGCGCCACATAGCAGAACTTTAAAAGTGCTCATCATTGGAAAACGTTCTTCGGG
pBluescript SK+ backbone
7,380 7,400 7,420 7,440
pYOGM081 map GCGAAAACTCTCAAGGATCTTACCGCTGTTGAGATCCAGTTCGATGTAACCCACTCGTGCACCCAACTGATCTTCAGCAT
pBluescript SK+ backbone
7,460 7,480 7,500 7,520
pYOGM081 map CTTTTACTTTCACCAGCGTTTCTGGGTGAGCAAAAACAGGAAGGCAAAATGCCGCAAAAAAGGGAATAAGGGCGACACGG
pBluescript SK+ backbone
7,540 7,560 7,580 7,600
pYOGM081 map AAATGTTGAATACTCATACTCTTCCTTTTTCAATATTATTGAAGCATTTATCAGGGTTATTGTCTCATGAGCGGATACAT
pBluescript SK+ backbone
7,620 7,640 7,660 7,680
pYOGM081 map ATTTGAATGTATTTAGAAAAATAAACAAATAGGGGTTCCGCGCACATTTCCCCGAAAAGTGCCACCTAAATTGTAAGCGT
pBluescript SK+ backbone
7,700 7,720 7,740 7,760
pYOGM081 map TAATATTTTGTTAAAATTCGCGTTAAATTTTTGTTAAATCAGCTCATTTTTTAACCAATAGGCCGAAATCGGCAAAATCC
pBluescript SK+ backbone
7,780 7,800 7,820 7,840
pYOGM081 map CTTATAAATCAAAAGAATAGACCGAGATAGGGTTGAGTGTTGTTCCAGTTTGGAACAAGAGTCCACTATTAAAGAACGTG
pBluescript SK+ backbone
7,860 7,880 7,900 7,920
pYOGM081 map GACTCCAACGTCAAAGGGCGAAAAACCGTCTATCAGGGCGATGGCCCACTACGTGAACCATCACCCTAATCAAGTTTTTT
pBluescript SK+ backbone
7,940 7,960 7,980 8,000
pYOGM081 map GGGGTCGAGGTGCCGTAAAGCACTAAATCGGAACCCTAAAGGGAGCCCCCGATTTAGAGCTTGACGGGGAAAGCCGGCGA
```

Figure S1 (continued).

```
pBluescript SK+ backbone
                 8,020     8,040     8,060     8,080
pYOGM081 map  ACGTGGCGAGAAAGGAAGGGAAGAAAGCGAAAGGAGCGGGCGCTAGGGCGCTGGCAAGTGTAGCGGTCACGCTGCGCGTA
pBluescript SK+ backbone
                 8,100     8,120     8,140     8,160
pYOGM081 map  ACCACCACACCCGCCGCGCTTAATGCGCCGCTACAGGGCGCGTCCCATTCGCCATTCAGGCTGCGCAACTGTTGGGAAGG
pBluescript SK+ backbone
                 8,180     8,200     8,220     8,240
pYOGM081 map  GCGATCGGTGCGGGCCTCTTCGCTATTACGCCAGCTGGCGAAAGGGGGATGTGCTGCAAGGCGATTAAGTTGGGTAACGC
pBluescript SK+ backbone
                 8,260     8,280     8,300     8,320
pYOGM081 map  CAGGGTTTTCCCAGTCACGACGTTGTAAAACGACGGCCAGTGAGCGCGCGTAATACGACTCACTATAGGGCGAATTGGGT
script SK+ backbone
pYOGM081 map  ACCGGGCCCCCC
```

***ADH1* promoter**

```
                 20     40     60     80
ADH1 promoter  ACAATATGGACTTCCTCTTTTCTGGCAACCAAACCCATACATCGGGATTCCTATAATACCTTCGTTGGTCTCCCTAACAT
                 100    120    140    160
ADH1 promoter  GTAGGTGGCGGAGGGGAGATATACAATAGAACAGATACCAGACAAGACATAATGGGCTAAACAAGACTACACCAATTACA
                 180    200    220    240
ADH1 promoter  CTGCCTCATTGATGGTGGTACATAACGAACTAATACTGTAGCCCTAGACTTGATAGCCATCATCATATCGAAGTTTCACT
                 260    280    300    320
ADH1 promoter  ACCCTTTTTCCATTTGCCATCTATTGAAGTAATAATAGGCGCATGCAACTTCTTTTCTTTTTTTTTCTTTTCTCTCTCCC
                 340    360    380    400
ADH1 promoter  CCGTTGTTGTCTCACCATATCCGCAATGACAAAAAAATGATGGAAGACGGGTGTACTAAAGGAAAAAATTAACGACAAAG
                 420    440    460    480
ADH1 promoter  ACAGCACCAACAGATGTCGTTGTTCCAGAGCTGATGAGGGGTATCTCGAAGCACACGAAACTTTTTCCTTCCTTCATTCA
                 500    520    540    560
ADH1 promoter  CGCACACTACTCTCTAATGAGCAACGGTATACGGCCTTCCTTCCAGTTACTTGAATTTGAAATAAAAAAAAGTTTGCTGT
                 580    600    620    640
ADH1 promoter  CTTGCTATCAAGTATAAATAGACCTGCAATTATTAATCTTTTGTTTCCTCGTCATTGTTCTCGTTCCCTTTCTTCCTTGT
                 660    680    700
ADH1 promoter  TTCTTTTTCTGCACAATATTTCAAGCTATACCAAGCATACAATCAACTATCTCATATACA
```

***TEF* promoter**

```
                20     40     60     80
TEF promoter  GACATGGAGGCCCAGAATACCCTCCTTGACAGTCTTGACGTGCGCAGCTCAGGGGCATGATGTGACTGTCGCCCGTACAT
                100    120    140    160
TEF promoter  TTAGCCCATACATCCCCATGTATAATCATTTGCATCCATACATTTTGATGGCCGCACGGCGCGAAGCAAAAATTACGGCT
                180    200    220    240
TEF promoter  CCTCGCTGCAGACCTGCGAGCAGGGAAACGCTCCCCTCACAGACGCGTTGAATTGTCCCCACGCCGCGCCCCTGTAGAGA
                260    280    300    320
TEF promoter  AATATAAAAGGTTAGGATTTGCCACTGAGGTTCTTCTTTCATATACTTCCTTTTAAAATCTTGCTAGGATACAGTTCTCA
                340
TEF promoter  CATCACATCCGAACATAAACAACC
```

Figure S1 (continued).

***CUP1* promoter**

```
                                       20                  40                  60                  80
CUP1 promoter  AACTTCAACGATTTCTATGATGCATTTTATAATTAGTAAGCCGATCCCATTACCGACATTTGGGCGCTATACGTGCATAT
                                      100                 120                 140                 160
CUP1 promoter  GTTCATGTATGTATCTGTATTTAAAACACTTTTGTATTATTTTTCCTCATATATGTGTATAGGTTTATACGGATGATTTA
                                      180                 200                 220                 240
CUP1 promoter  ATTATTACTTCACCACCCTTTATTTCAGGCTGATATCTTAGCCTTGTTACTAGTTAGAAAAAGACATTTTTGCTGTCAGT
                                      260                 280                 300                 320
CUP1 promoter  CACTGTCAAGAGATTCTTTTGCTGGCATTTCTTCTAGAAGCAAAAAGAGCGATGCGTCTTTTCCGCTGAACCGTTCCAGC
                                      340                 360                 380                 400
CUP1 promoter  AAAAAAGACTACCAACGCAATATGGATTGTCAGAATCATATAAAAGAGAAGCAAATAACTCCTTGTCTTGTATCAATTGC
                                      420                 440                 460                 480
CUP1 promoter  ATTATAATATCTTCTTGTTAGTGCAATATCATATAGAAGTCATCGAAATAGATATTAAGAAAAACAAACTGTACAATCAA
                                      500
CUP1 promoter  TCAATCAATCATCACATAAA
```

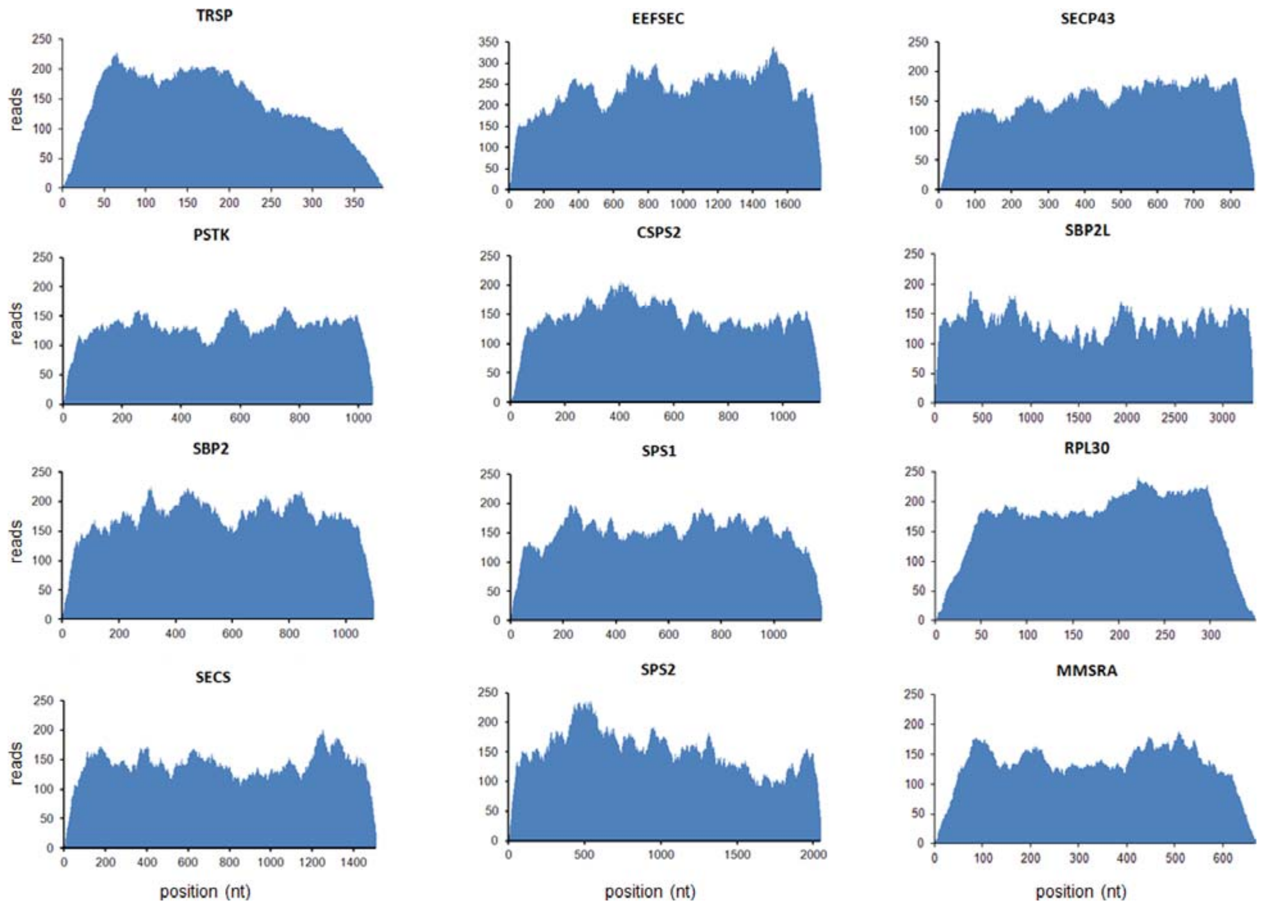

Figure S2 Integration of the metazoan Sec biosynthesis and insertion genes into the yeast genome. Panels show read coverage of exogenous genes introduced using iGM method. Coverage density maps were generated by aligning reads obtained by sequencing the whole genome of the iGM11 mutant to the sequences of the introduced exogenous genes. The entire ORF of the gene beginning from the ATG start codon and ending at the stop codon were used for alignment. For SPS2, which is a selenoprotein, its ORF together with 3'-UTR flanking region containing a SECIS element was used to generate the coverage map.

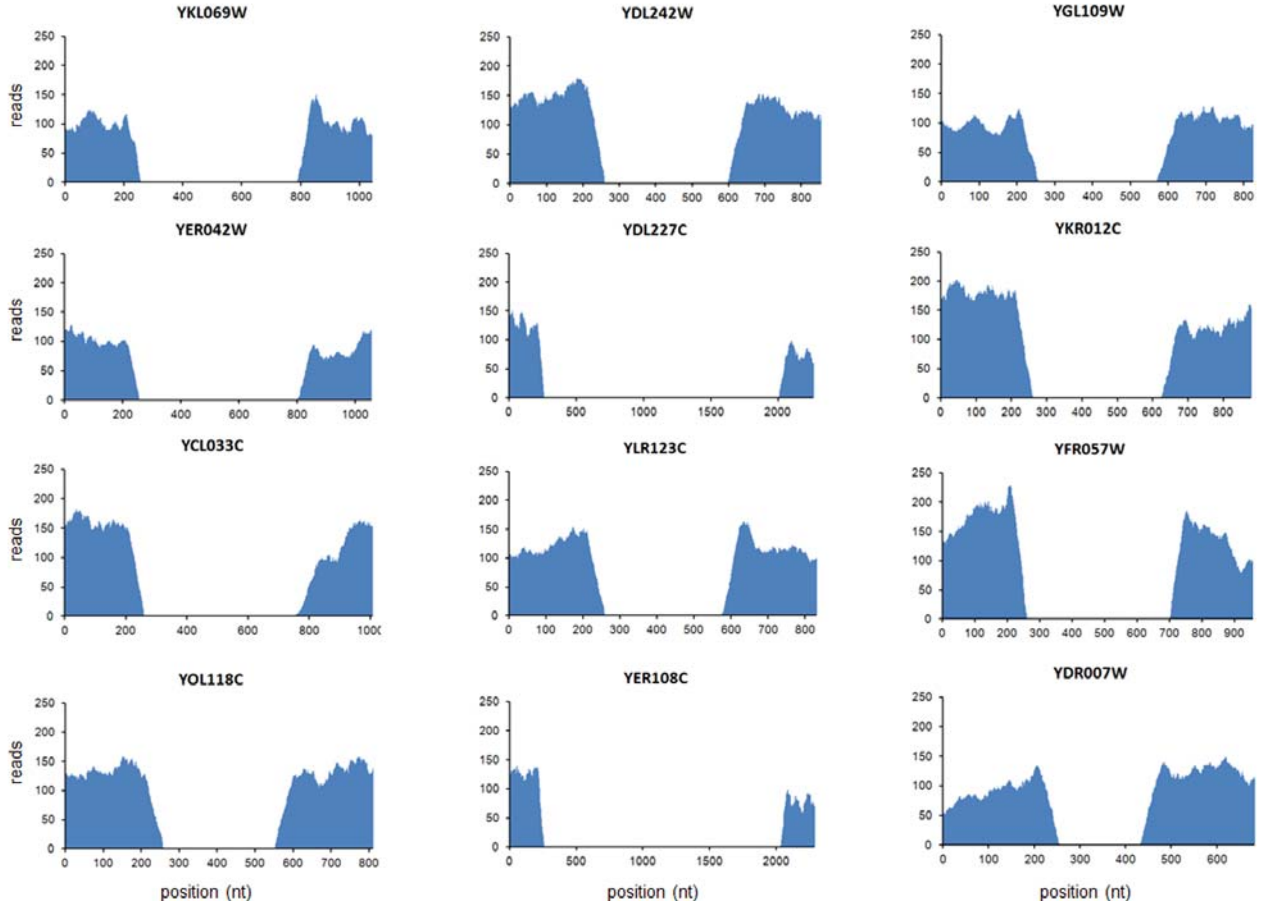

Figure S3 Sequencing of the whole genome confirms deletion of the yeast genes. Panels show read coverage of endogenous genes that were replaced by inserted genes. Coverage density maps were generated by aligning reads obtained by sequencing the whole genome of the iGM11 mutant to the sequences of the deleted genes together with 250 bp 5'-UTR and 250 bp 3'-UTR flanking regions.

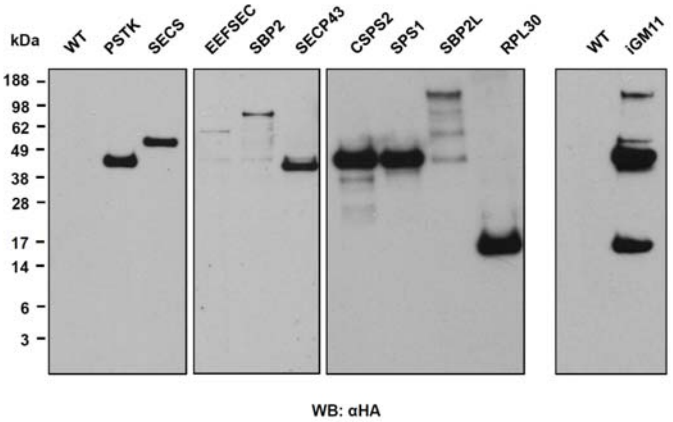

Expected size (kDa)	
SBP2L	128.0
SBP2	101.5
EEFSEC	71.4
SECS	61.8
SPS1	49.1
CSPS2	47.6
PSTK	45.7
SECP43	38.7
RPL30	18.9

Figure S4 Expression analysis of the exogenous genes introduced into the yeast genome using iGM method. Expression of proteins carrying HA-tag upon culture of cells in the presence of galactose was detected in individual ProMonster strains and a strain containing all of the 11 gene insertions (iGM11) by Western blotting with HA-tag specific antibodies. Expected sizes of proteins are shown on the right.

Table S1 PCR primers used for generating a universal gene insertion module.

Primer sequence	Template
**Fragment 1**	
**1st round**<br>5'-CAGAAGCTT(HindIII)TCAGTACTGACAATAAAAAGATTC-3'<br>5'-TAGAGCTC(SacI)GCTAGC(NheI)GGCGCGCCAGATCT(BglII) GAGCTC(SacI)GTTTTCGACAC-3'<br>**2nd round**<br>5'-CAGAAGCTT(HindIII)TCAGTACTGACAATAAAAAGATTC-3'<br>5'-GGAATTC(EcoRI)ACCAGGT(SexAI)CCGCGG(SacII)TTAATTA A(PacI)TACGTA(SnaBI)GAGCTC(SacI)GCTAG-3'	Template for the first round of PCR: pAG25 (Goldstein and McCusker 1999)<br>To extend the downstream end, the product of the first round of PCR was used as a template for the second round of PCR
**Fragment 2**	
5'-CCGCTCGAG(XhoI)CTAGC(NheI)CGCGG(SacII)TACGTA(SnaBI) CCAGGT(SexAI)GAGCTC(SacI)GACGGATCCCCGGGTTAA-3'<br>5'-CCCAAGCTT(HindIII)GGGATATCGCGTCGAC(SalI)GCACGTC AAGACTGTCAAGG-3'	pAG25 (Goldstein and McCusker 1999)
**Fragment 3**	
5'-CCGCTCGAG(XhoI)CTTTTCAATTCAATTCATC-3'<br>5'-CCCAAGCTT(HindIII)GGGATATCGCGTCGAC(SalI)GGGTAAT AACTGATATAAT-3'	pYOGM057 with the *URA3* sequence derived from pRS316 (Sikorski and Hieter 1989)
**Fragment 4**	
PCR was not used for this fragment	
**Fragment 5**	
5'-GAAGGCCT(StuI)GTACGGATTAGAAGCCGCCGAG-3'<br>5'-GAAGGCCT(StuI)GACTCGAGTTAGCACTGAGC-3'	pAG416GAL-ccdB-HA (Alberti *et al.* 2007)
**HA-less construct**	
HAless-HindIII_F 5'-GACTAAGCTT(HindIII)TCAGTACTGACAATAAAAAGATTCTTG-3'<br>HAless-HindIII_R 5'-GACTAAGCTT(HindIII)TTA(Stop)GCAGCCCATCACCACTTTG-3'	pYOGM081
**Construct with the *ADH1* promoter**	
ADH1pr_Fwd 5'-TGCGGCCAAGCTCCTGTACAATATGGACTTCCTCTTTTCTG-3'<br>ADH1pr_Rev 5'-GCTTTTTTGTACAAACTTGTGATTGTATATGAGATAGTTGATTG-3'	BY4741 genomic DNA
ADH1pr_pYOGM081_Fwd 5'-CTATCTCATATACAATCACAAGTTTGTACAAAAAAGCTGAACG-3'<br>ADH1pr_pYOGM081_Rev 5'-GAGGAAGTCCATATTGTACAGGAGCTTGGCCGCAAATTAAAG-3'	pYOGM081
**Construct with the *TEF* promoter**	
TEFpr_Fwd 5'-TGCGGCCAAGCTCCTGTGACATGGAGGCCCAGAATACCCTC-3'<br>TEFpr_Rev 5'-GCTTTTTTGTACAAACTTGTGATGGTTGTTTATGTTCGGATGTG-3'	pFA6a-kanMX4 (Wach *et al.* 1994)
TEFpr_pYOGM081_Fwd 5'-CCGAACATAAACAACCATCACAAGTTTGTACAAAAAAGCTGAAC-3'<br>TEFpr_pYOGM081_Rev 5'-CTGGGCCTCCATGTCACAGGAGCTTGGCCGCAAATTAAAGCC-3'	pYOGM081
**Construct with the *CUP1* promoter**	
CUP1pr_Fwd 5'-GCTTTAATTTGCGGCCAAGCTCCTGTAACTTCAACGATTTCTATGATGC-3'<br>CUP1pr_Rev 5'-CAGCTTTTTTGTACAAACTTGTGATTTTATGTGATGATTGATTG-3'	BY4741 genomic DNA
CUP1pr_pYOGM081_Fwd 5'-CAATCATCACATAAAATCACAAGTTTGTACAAAAAAGC TGAACGAGAAACG-3'<br>CUP1pr_pYOGM081_Rev 5'-CATAGAAATCGTTGAAGTTACAGGAGCTTGGCCGCAAA TTAAAGCCTTC-3'	pYOGM081

Table S2 List of genotyping primers used in this study.

Deleted ORF	Gene insertion	Primer sequence
YKL069W	TRSP	5’-TAGCGACAGAGTGGTTCAATTC-3’<br>5’-TCAATTTGGCGAACAGGGAATG-3’
YER042W	PSTK	5’-AGTGTTGCAGAATCGAGAAGAG-3’<br>5’-TCATAAATAAGGGCACGTACAC-3’
YOL118C	SECS	5’-ACACATACCAGGATGCTTCTTC-3’<br>5’-GCTGACTAATTTGAAGCTATCG-3’
YLR123C	SPS1	5’-ACAGCCAGAATCATAGACAAAC-3’<br>5’-TTCAGCTGATGTGCCATGTAAC-3’
YER108C	SPS2	5’-GATGTTAAGTCTTTTGCGGCAG-3’<br>5’-AAAGTCGTTGCTGTGAAAATGG-3’
YCL033C	SBP2	5’-AAGAATCCTTGGAGGCTTCAAC-3’<br>5’-GTCCACGATCTCAAACCCTTTC-3’
YKR012C	SBP2L	5’-TTACACAACGCAAACTACGTAC-3’<br>5’-TTAGGACCATCTTGCATTTGAG-3’
YDL242W	EEFSEC	5’-TCAAGCGTTATGTCTTCGACAC-3’<br>5’-GTTTCGATATTCGCACATTTGC-3’
YGL109W	SECP43	5’-ACAAGGAGTTCATGGAACAGAG-3’<br>5’-CAACTAAAGAGTACAACTGTCC-3’
YFR057W	RPL30	5’-CTCTGACATCATTAGAAGCATG-3’<br>5’-AGATAACTCTGAACTGTGCATC-3’
YDL227C	cSPS2	5’-CTCTGTTCCCTCTCATATTTAC-3’<br>5’-CTACTCCAGCATTCTAGTTAAG-3’

SUPPLEMENTARY REFERENCES

Alberti, S., A. D. Gitler, and S. Lindquist, 2007 A suite of Gateway cloning vectors for high-throughput genetic analysis in Saccharomyces cerevisiae. Yeast 24: 913-919.
Goldstein, A. L., and J. H. McCusker, 1999 Three new dominant drug resistance cassettes for gene disruption in Saccharomyces cerevisiae. Yeast 15: 1541-1553.
Sikorski, R. S., and P. Hieter, 1989 A system of shuttle vectors and yeast host strains designed for efficient manipulation of DNA in Saccharomyces cerevisiae. Genetics 122: 19-27.
Wach, A., A. Brachat, R. Pohlmann, and P. Philippsen, 1994 New heterologous modules for classical or PCR-based gene disruptions in Saccharomyces cerevisiae. Yeast 10: 1793-1808.
