# Supplementary material for: The Insertion Green Monster (iGM) Method for Expression of Multiple Exogenous Genes in Yeast
Source: G3 (Bethesda). 2014 Apr 28;4(7):1183–91. doi: 10.1534/g3.114.010868 (PMC4455768; doi:10.1534/g3.114.010868)
Supplement: Supporting Information [file supp_g3.114.010868_FigureS1.pdf]

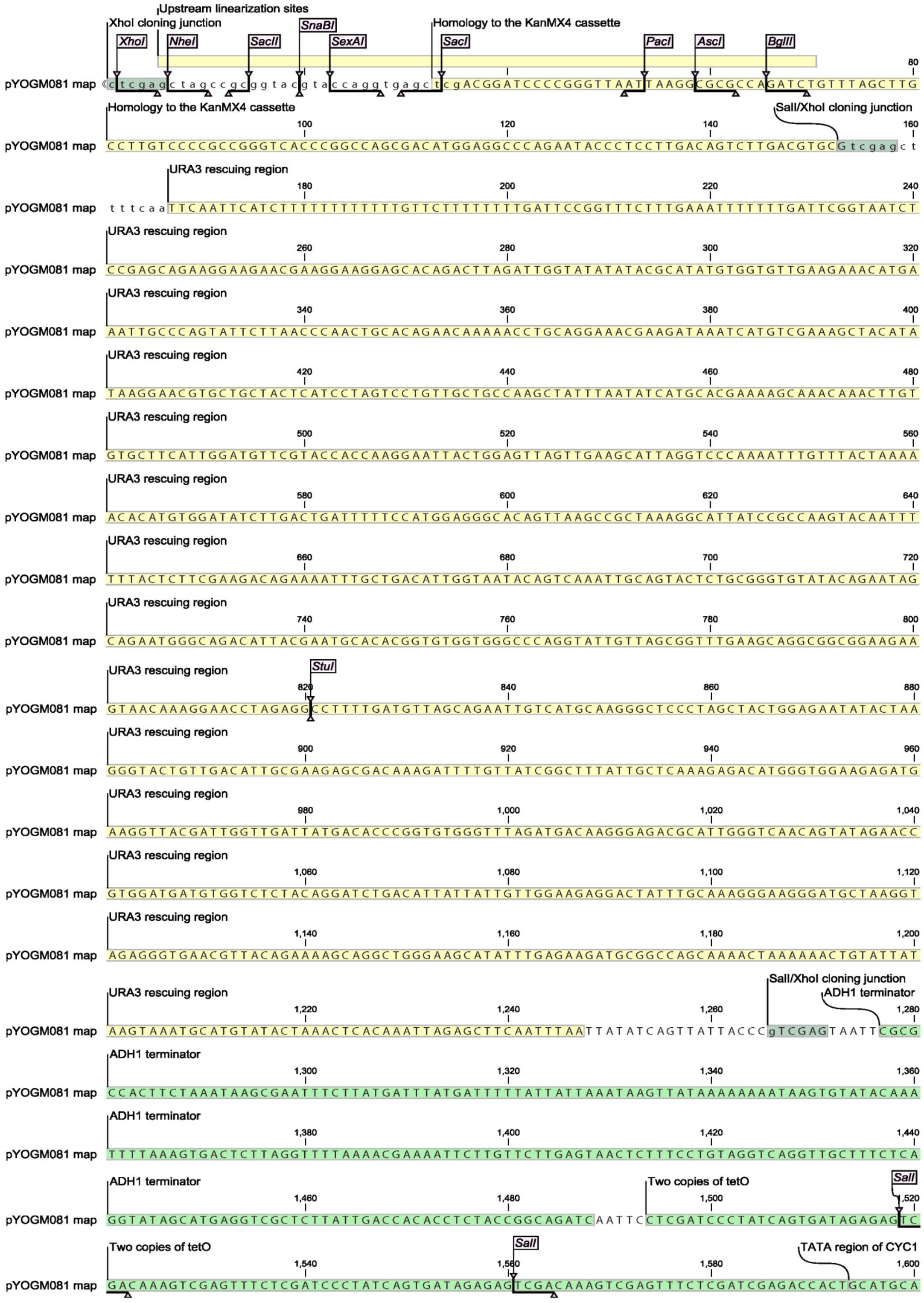

Figure S1 Sequence of the pYOGM081 plasmid containing the iGM gene insertion module. In a version of the plasmid not containing the HA-tag (pTJH001), nucleotides 4,954–5,101 are removed and replaced with a stop codon (TAA) and a *Hin*dIII site (Materials and Methods). In plasmids containing the *ADH1* (pTJH002), *TEF* (pTJH003), and *CUP1* (pTJH004) promoters for driving the expression of a gene introduced between the Gateway cloning sites, nucleotides 2,755–3,232 are replaced with the respective sequences shown below (Materials and Methods).

Figure S1 (continued).

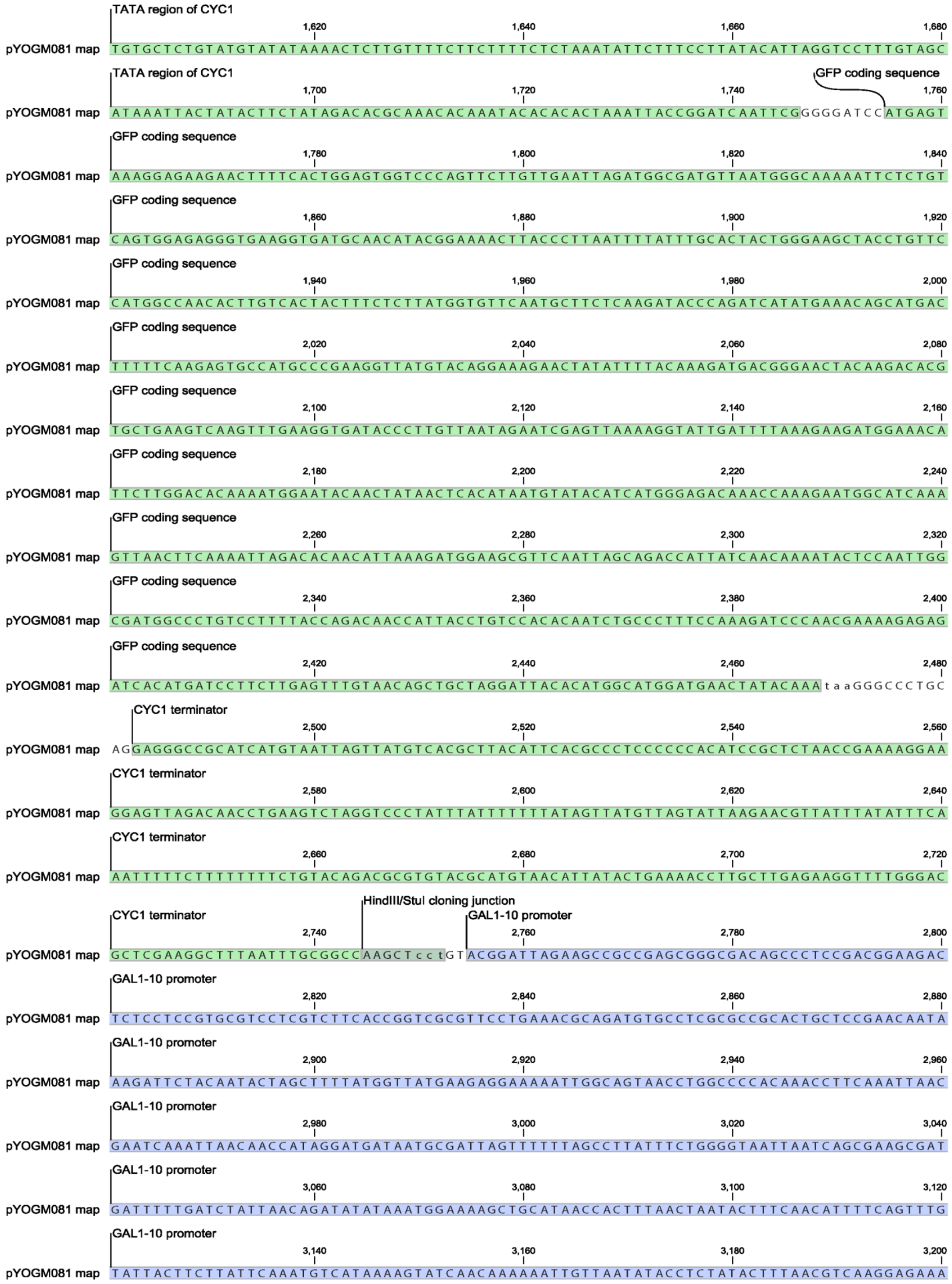

Figure S1 (continued).

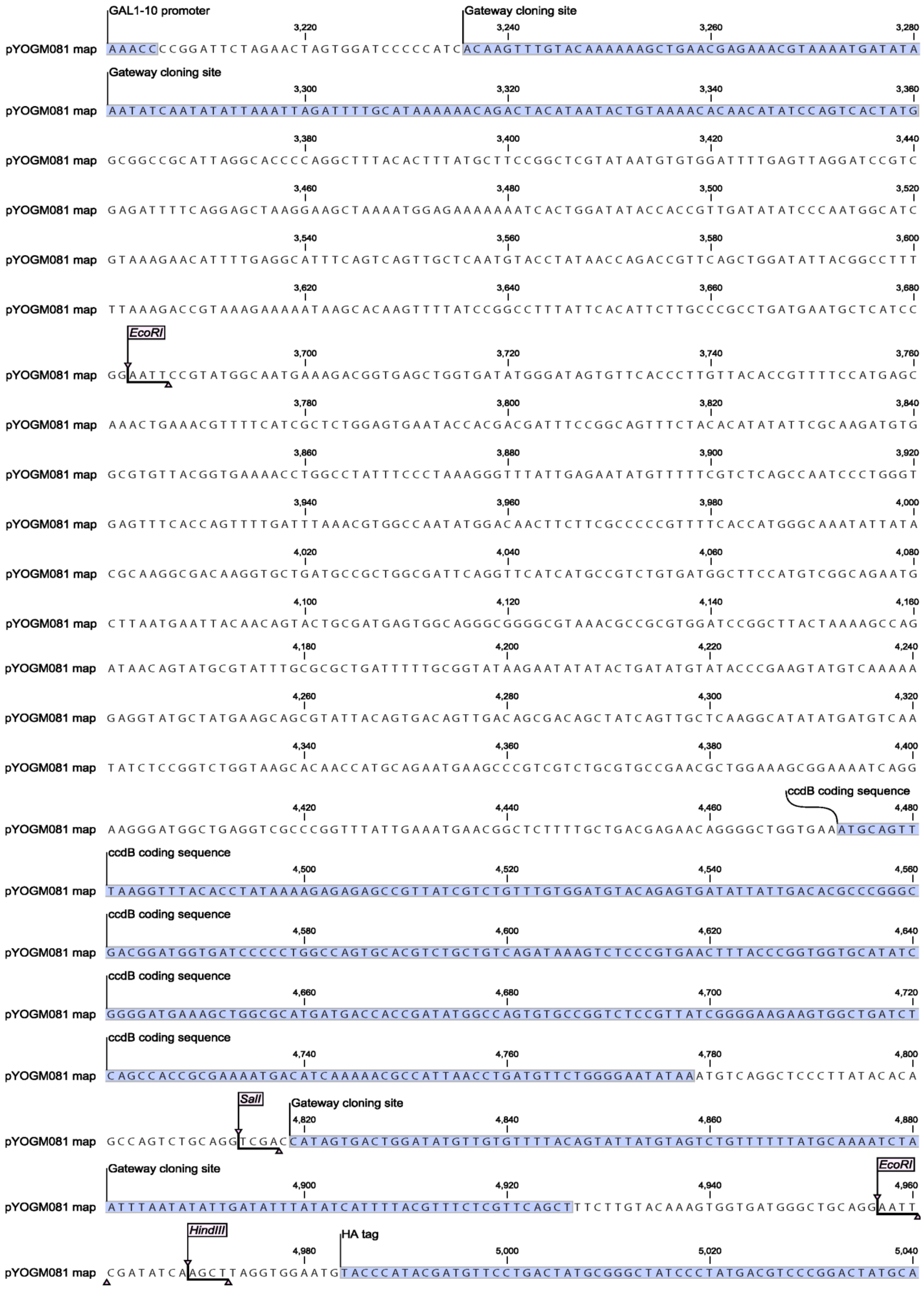

Figure S1 (continued).

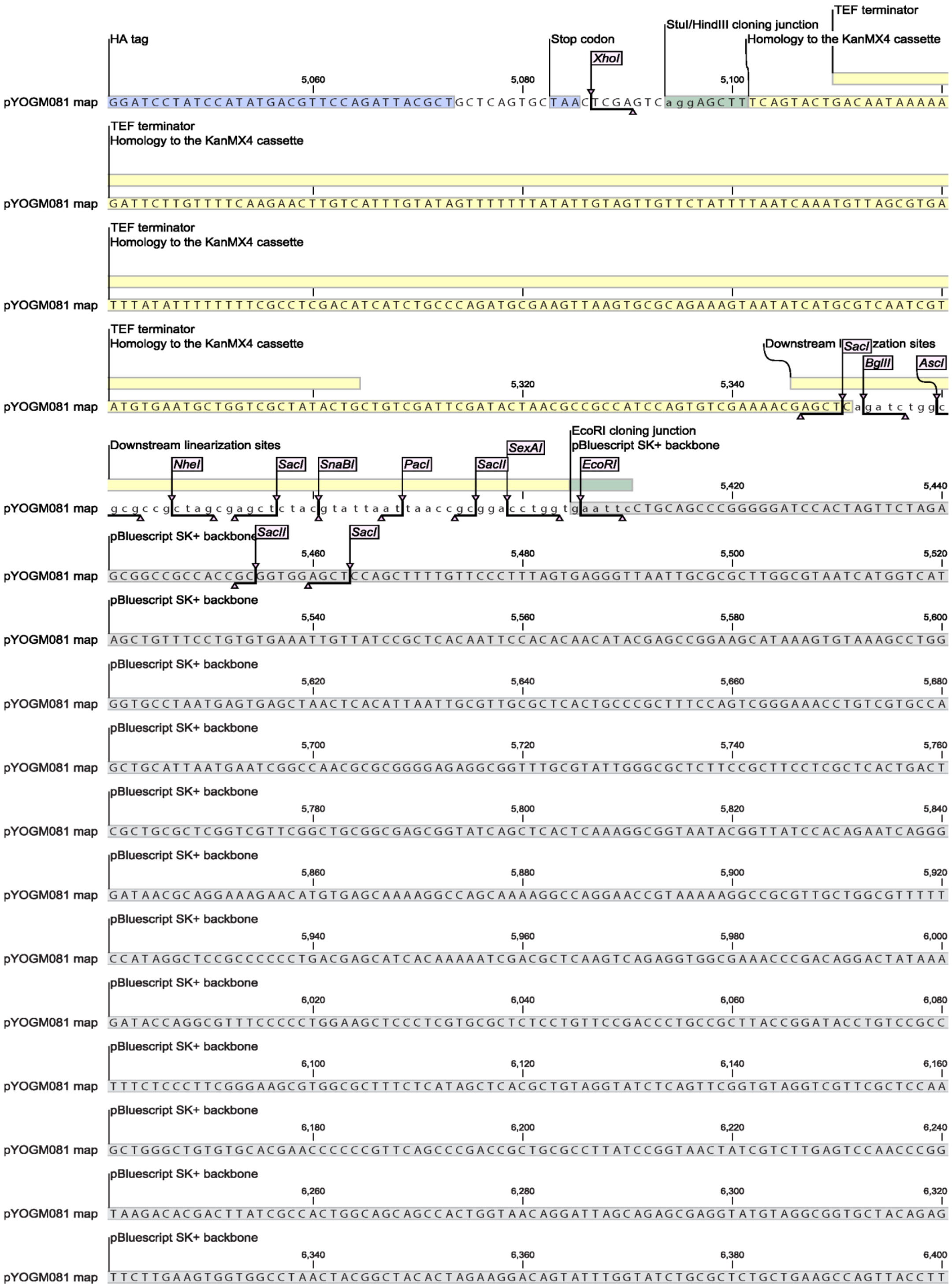

Figure S1 (continued).

pBluescript SK+ backbone
6,420 6,440 6,460 6,480
pYOGM081 map CGGAAAAAGAGTTGGTAGCTCTTGATCCGGCAAACAAACCACCGCTGGTAGCGGTGGTTTTTTTGTTTGCAAGCAGCAGA

pBluescript SK+ backbone
6,500 6,520 6,540 6,560
pYOGM081 map TTACGCGCAGAAAAAAAGGATCTCAAGAAGATCCTTTGATCTTTTCTACGGGGTCTGACGCTCAGTGGAACGAAAACTCA

pBluescript SK+ backbone
6,580 6,600 6,620 6,640
pYOGM081 map CGTTAAGGGATTTTGGTCATGAGATTATCAAAAAGGATCTTCACCTAGATCCTTTTAAATTAAAAATGAAGTTTTAAATC

pBluescript SK+ backbone
6,660 6,680 6,700 6,720
pYOGM081 map AATCTAAAGTATATATGAGTAAACTTGGTCTGACAGTTACCAATGCTTAATCAGTGAGGCACCTATCTCAGCGATCTGTC

pBluescript SK+ backbone
6,740 6,760 6,780 6,800
pYOGM081 map TATTTCGTTCATCCATAGTTGCCTGACTCCCCGTCGTGTAGATAACTACGATACGGGAGGGCTTACCATCTGGCCCCAGT

pBluescript SK+ backbone
6,820 6,840 6,860 6,880
pYOGM081 map GCTGCAATGATACCGCGAGACCCACGCTCACCGGCTCCAGATTTATCAGCAATAAACCAGCCAGCCGGAAGGGCCGAGCG

pBluescript SK+ backbone
6,900 6,920 6,940 6,960
pYOGM081 map CAGAAGTGGTCCTGCAACTTTATCCGCCTCCATCCAGTCTATTAATTGTTGCCGGGAAGCTAGAGTAAGTAGTTCGCCAG

pBluescript SK+ backbone
6,980 7,000 7,020 7,040
pYOGM081 map TTAATAGTTTGCGCAACGTTGTTGCCATTGCTACAGGCATCGTGGTGTCACGCTCGTCGTTTGGTATGGCTTCATTCAGC

pBluescript SK+ backbone
7,060 7,080 7,100 7,120
pYOGM081 map TCCGGTTCCCAACGATCAAGGCGAGTTACATGATCCCCCATGTTGTGCAAAAAAGCGGTTAGCTCCTTCGGTCCTCCGAT

pBluescript SK+ backbone
7,140 7,160 7,180 7,200
pYOGM081 map CGTTGTCAGAAGTAAGTTGGCCGCAGTGTTATCACTCATGGTTATGGCAGCACTGCATAATTCTCTTACTGTCATGCCAT

pBluescript SK+ backbone
7,220 7,240 7,260 7,280
pYOGM081 map CCGTAAGATGCTTTTCTGTGACTGGTGAGTACTCAACCAAGTCATTCTGAGAATAGTGTATGCGGCGACCGAGTTGCTCT

pBluescript SK+ backbone
7,300 7,320 7,340 7,360
pYOGM081 map TGCCCGGCGTCAATACGGGATAATACCGCGCCACATAGCAGAACTTTAAAAGTGCTCATCATTGGAAAACGTTCTTCGGG

pBluescript SK+ backbone
7,380 7,400 7,420 7,440
pYOGM081 map GCGAAAACTCTCAAGGATCTTACCGCTGTTGAGATCCAGTTCGATGTAACCCACTCGTGCACCCAACTGATCTTCAGCAT

pBluescript SK+ backbone
7,460 7,480 7,500 7,520
pYOGM081 map CTTTTACTTTCACCAGCGTTTCTGGGTGAGCAAAAACAGGAAGGCAAAATGCCGCAAAAAAGGGAATAAGGGCGACACGG

pBluescript SK+ backbone
7,540 7,560 7,580 7,600
pYOGM081 map AAATGTTGAATACTCATACTCTTCCTTTTTCAATATTATTGAAGCATTTATCAGGGTTATTGTCTCATGAGCGGATACAT

pBluescript SK+ backbone
7,620 7,640 7,660 7,680
pYOGM081 map ATTTGAATGTATTTAGAAAAATAAACAAATAGGGGTTCCGCGCACATTTCCCCGAAAAGTGCCACCTAAATTGTAAGCGT

pBluescript SK+ backbone
7,700 7,720 7,740 7,760
pYOGM081 map TAATATTTTGTTAAAATTCGCGTTAAATTTTTGTTAAATCAGCTCATTTTTTAACCAATAGGCCGAAATCGGCAAAATCC

pBluescript SK+ backbone
7,780 7,800 7,820 7,840
pYOGM081 map CTTATAAATCAAAAGAATAGACCGAGATAGGGTTGAGTGTTGTTCCAGTTTGGAACAAGAGTCCACTATTAAAGAACGTG

pBluescript SK+ backbone
7,860 7,880 7,900 7,920
pYOGM081 map GACTCCAACGTCAAAGGGCGAAAAACCGTCTATCAGGGCGATGGCCCACTACGTGAACCATCACCCTAATCAAGTTTTTT

pBluescript SK+ backbone
7,940 7,960 7,980 8,000
pYOGM081 map GGGGTCGAGGTGCCGTAAAGCACTAAATCGGAACCCTAAAGGGAGCCCCCGATTTAGAGCTTGACGGGGAAAGCCGGCGA

Figure S1 (continued).

pBluescript SK+ backbone
8,020 8,040 8,060 8,080
pYOGM081 map ACGTGGCGAGAAAGGAAGGGAAGAAAGCGAAAGGAGCGGGCGCTAGGGCGCTGGCAAGTGTAGCGGTCACGCTGCGCGTA

pBluescript SK+ backbone
8,100 8,120 8,140 8,160
pYOGM081 map ACCACCACACCCGCCGCGCTTAATGCGCCGCTACAGGGCGCGTCCCATTCGCCATTCAGGCTGCGCAACTGTTGGGAAGG

pBluescript SK+ backbone
8,180 8,200 8,220 8,240
pYOGM081 map GCGATCGGTGCGGGCCTCTTCGCTATTACGCCAGCTGGCGAAAGGGGGATGTGCTGCAAGGCGATTAAGTTGGGTAACGC

pBluescript SK+ backbone
8,260 8,280 8,300 8,320
pYOGM081 map CAGGGTTTTCCCAGTCACGACGTTGTAAAACGACGGCCAGTGAGCGCGCGTAATACGACTCACTATAGGGCGAATTGGGT

escript SK+ backbone
pYOGM081 map ACCGGGCCCCCC

ADH1 promoter

20 40 60 80
ADH1 promoter ACAATATGGACTTCCTCTTTTCTGGCAACCAAACCCATACATCGGGATTCCTATAATACCTTCGTTGGTCTCCCTAACAT

100 120 140 160
ADH1 promoter GTAGGTGGCGGAGGGGAGATATACAATAGAACAGATACCAGACAAGACATAATGGGCTAAACAAGACTACACCAATTACA

180 200 220 240
ADH1 promoter CTGCCTCATTGATGGTGGTACATAACGAACTAATACTGTAGCCCTAGACTTGATAGCCATCATCATATCGAAGTTTCACT

260 280 300 320
ADH1 promoter ACCCTTTTTCCATTTGCCATCTATTGAAGTAATAATAGGCGCATGCAACTTCTTTTCTTTTTTTTTCTTTTCTCTCTCCC

340 360 380 400
ADH1 promoter CCGTTGTTGTCTCACCATATCCGCAATGACAAAAAAATGATGGAAGACGGGTGTACTAAAGGAAAAAATTAACGACAAAG

420 440 460 480
ADH1 promoter ACAGCACCAACAGATGTCGTTGTTCCAGAGCTGATGAGGGGTATCTCGAAGCACACGAAACTTTTTCCTTCCTTCATTCA

500 520 540 560
ADH1 promoter CGCACACTACTCTCTAATGAGCAACGGTATACGGCCTTCCTTCCAGTTACTTGAATTTGAAATAAAAAAAAGTTTGCTGT

580 600 620 640
ADH1 promoter CTTGCTATCAAGTATAAATAGACCTGCAATTATTAATCTTTTGTTTCCTCGTCATTGTTCTCGTTCCCTTTCTTCCTTGT

660 680 700
ADH1 promoter TTCTTTTTCTGCACAATATTTCAAGCTATACCAAGCATACAATCAACTATCTCATATACA

TEF promoter

20 40 60 80
TEF promoter GACATGGAGGCCCAGAATACCCTCCTTGACAGTCTTGACGTGCGCAGCTCAGGGGCATGATGTGACTGTCGCCCGTACAT

100 120 140 160
TEF promoter TTAGCCCATACATCCCCATGTATAATCATTTGCATCCATACATTTTGATGGCCGCACGGCGCGAAGCAAAAATTACGGCT

180 200 220 240
TEF promoter CCTCGCTGCAGACCTGCGAGCAGGGAAACGCTCCCCTCACAGACGCGTTGAATTGTCCCCACGCCGCGCCCCTGTAGAGA

260 280 300 320
TEF promoter AATATAAAAGGTTAGGATTTGCCACTGAGGTTCTTCTTTCATATACTTCCTTTTAAAATCTTGCTAGGATACAGTTCTCA

340
TEF promoter CATCACATCCGAACATAAACAACC

Figure S1 (continued).

***CUP1* promoter**

CUP1 promoter	AACTTCAACGATTTCTATGATGCATTTTATAATTAGTAAGCCGATCCCATTACCGACATTTGGGCGCTATACGTGCATAT	80
CUP1 promoter	GTTCATGTATGTATCTGTATTTAAAACACTTTTGTATTATTTTTCCTCATATATGTGTATAGGTTTATACGGATGATTTA	160
CUP1 promoter	ATTATTACTTCACCACCCTTTATTTCAGGCTGATATCTTAGCCTTGTTACTAGTTAGAAAAAGACATTTTTGCTGTCAGT	240
CUP1 promoter	CACTGTCAAGAGATTCTTTTGCTGGCATTTCTTCTAGAAGCAAAAAGAGCGATGCGTCTTTTCCGCTGAACCGTTCCAGC	320
CUP1 promoter	AAAAAAGACTACCAACGCAATATGGATTGTCAGAATCATATAAAAGAGAAGCAAATAACTCCTTGTCTTGTATCAATTGC	400
CUP1 promoter	ATTATAATATCTTCTTGTTAGTGCAATATCATATAGAAGTCATCGAAATAGATATTAAGAAAAACAAACTGTACAATCAA	480
CUP1 promoter	TCAATCAATCATCACATAAA	500
