# Supplementary material for: The Insertion Green Monster (iGM) Method for Expression of Multiple Exogenous Genes in Yeast
Source: G3 (Bethesda). 2014 Apr 28;4(7):1183–91. doi: 10.1534/g3.114.010868 (PMC4455768; doi:10.1534/g3.114.010868)
Supplement: Supporting Information [file supp_g3.114.010868_FigureS2.pdf]

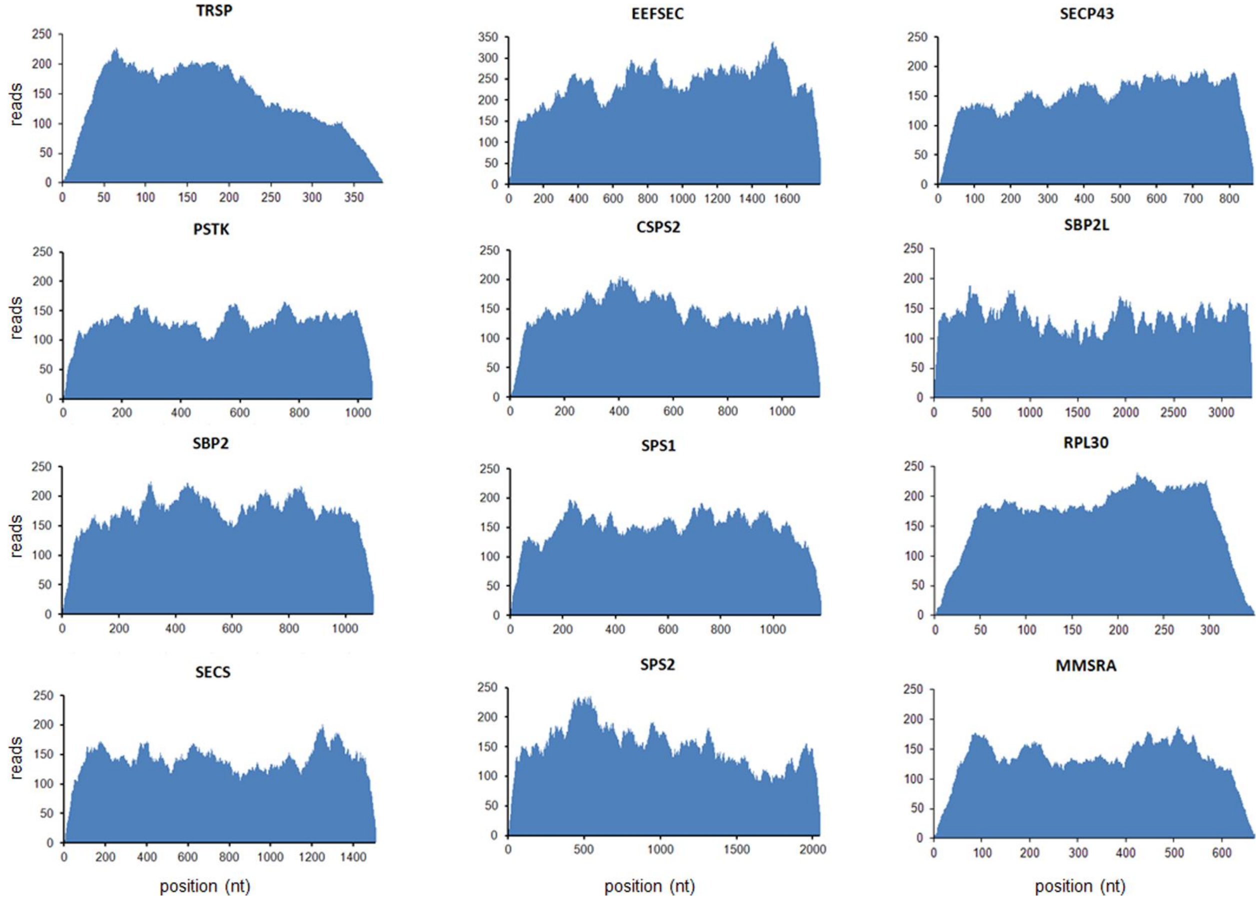

Figure S2 Integration of the metazoan Sec biosynthesis and insertion genes into the yeast genome. Panels show read coverage of exogenous genes introduced using iGM method. Coverage density maps were generated by aligning reads obtained by sequencing the whole genome of the iGM11 mutant to the sequences of the introduced exogenous genes. The entire ORF of the gene beginning from the ATG start codon and ending at the stop codon were used for alignment. For SPS2, which is a selenoprotein, its ORF together with 3'-UTR flanking region containing a SECIS element was used to generate the coverage map.
