# Supplementary material for: The Insertion Green Monster (iGM) Method for Expression of Multiple Exogenous Genes in Yeast
Source: G3 (Bethesda). 2014 Apr 28;4(7):1183–91. doi: 10.1534/g3.114.010868 (PMC4455768; doi:10.1534/g3.114.010868)
Supplement: Supporting Information [file supp_g3.114.010868_FigureS3.pdf]

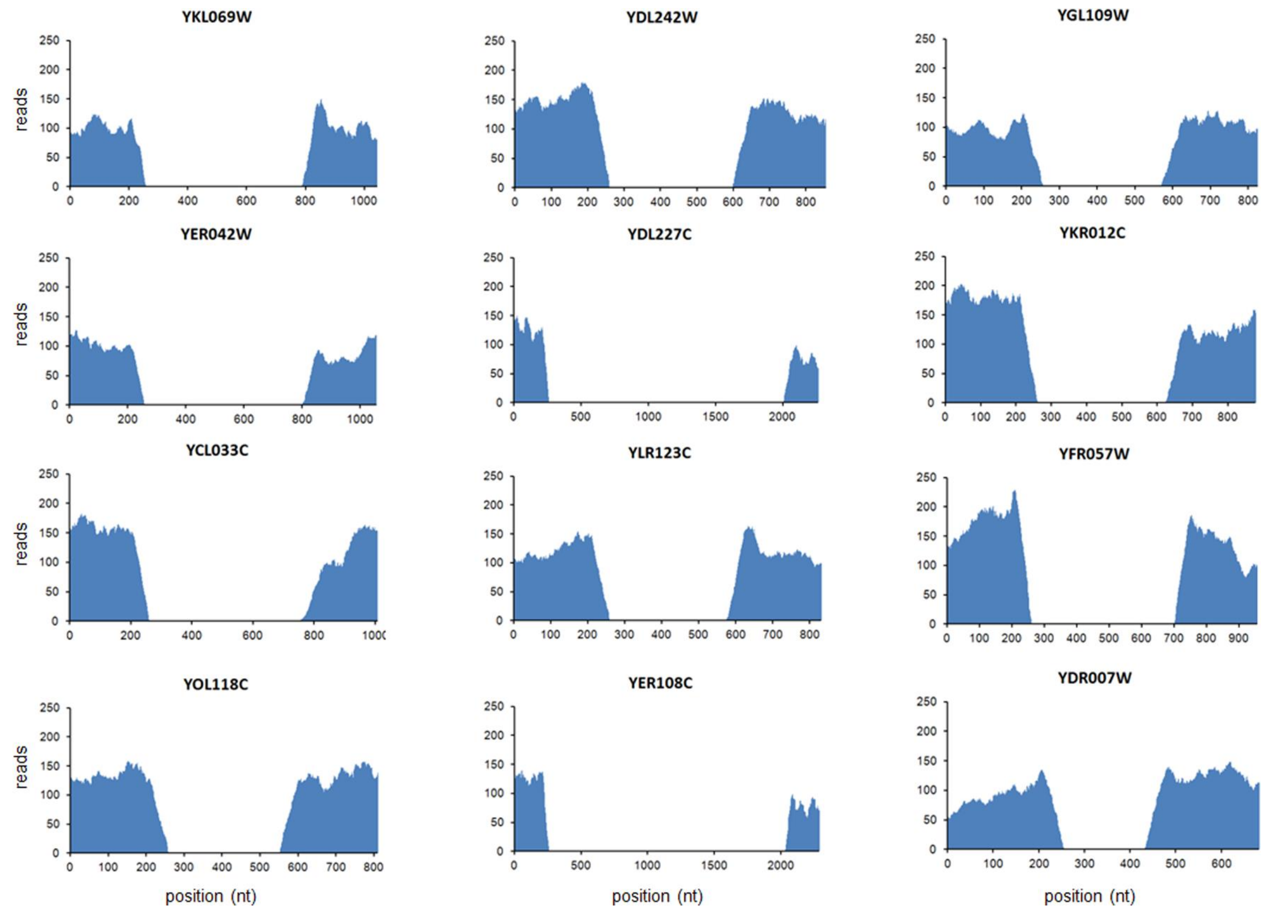

Figure S3 Sequencing of the whole genome confirms deletion of the yeast genes. Panels show read coverage of endogenous genes that were replaced by inserted genes. Coverage density maps were generated by aligning reads obtained by sequencing the whole genome of the iGM11 mutant to the sequences of the deleted genes together with 250 bp 5'-UTR and 250 bp 3'-UTR flanking regions.
