# Supplementary material for: The Insertion Green Monster (iGM) Method for Expression of Multiple Exogenous Genes in Yeast
Source: G3 (Bethesda). 2014 Apr 28;4(7):1183–91. doi: 10.1534/g3.114.010868 (PMC4455768; doi:10.1534/g3.114.010868)
Supplement: Supporting Information [file supp_g3.114.010868_FigureS4.pdf]

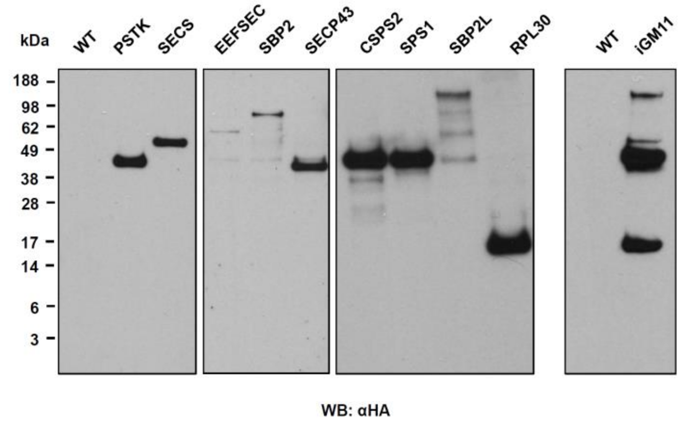

Expected size (kDa)

SBP2L	128.0
SBP2	101.5
EEFSEC	71.4
SECS	61.8
SPS1	49.1
CSPS2	47.6
PSTK	45.7
SECP43	38.7
RPL30	18.9

Figure S4 Expression analysis of the exogenous genes introduced into the yeast genome using iGM method. Expression of proteins carrying HA-tag upon culture of cells in the presence of galactose was detected in individual ProMonster strains and a strain containing all of the 11 gene insertions (iGM11) by Western blotting with HA-tag specific antibodies. Expected sizes of proteins are shown on the right.
