# Supplementary material for: The Insertion Green Monster (iGM) Method for Expression of Multiple Exogenous Genes in Yeast
Source: G3 (Bethesda). 2014 Apr 28;4(7):1183–91. doi: 10.1534/g3.114.010868 (PMC4455768; doi:10.1534/g3.114.010868)
Supplement: Supporting Information [file supp_g3.114.010868_TableS1.pdf]

Table S1 PCR primers used for generating a universal gene insertion module.

Primer sequence	Template
**Fragment 1**	
**1st round**<br>5'-CAGAAGCTT(HindIII)TCAGTACTGACAATAAAAAGATTC-3'<br>5'-TAGAGCTC(SacI)GCTAGC(NheI)GGCGCGCCAGATCT(BglII) GAGCTC(SacI)GTTTTCGACAC-3'<br><br>**2nd round**<br>5'-CAGAAGCTT(HindIII)TCAGTACTGACAATAAAAAGATTC-3'<br>5'-GGAATTC(EcoRI)ACCAGGT(SexAI)CCGCGG(SacII)TTAATTA A(PacI)TACGTA(SnaBI)GAGCTC(SacI)GCTAG-3'	Template for the first round of PCR: pAG25 (Goldstein and McCusker 1999)<br><br>To extend the downstream end, the product of the first round of PCR was used as a template for the second round of PCR
**Fragment 2**	
5'-CCGCTCGAG(XhoI)CTAGC(NheI)CGCGG(SacII)TACGTA(SnaBI) CCAGGT(SexAI)GAGCTC(SacI)GACGGATCCCCGGGTTAA-3'<br>5'-CCCAAGCTT(HindIII)GGGATATCGCGTCGAC(SalI)GCACGTC AAGACTGTCAAGG-3'	pAG25 (Goldstein and McCusker 1999)
**Fragment 3**	
5'-CCGCTCGAG(XhoI)CTTTTCAATTCAATTCATC-3'<br>5'-CCCAAGCTT(HindIII)GGGATATCGCGTCGAC(SalI)GGGTAAT AACTGATATAAT-3'	pYOGM057 with the *URA3* sequence derived from pRS316 (Sikorski and Hieter 1989)
**Fragment 4**	
PCR was not used for this fragment	
**Fragment 5**	
5'-GAAGGCCT(StuI)GTACGGATTAGAAGCCGCCGAG-3'<br>5'-GAAGGCCT(StuI)GACTCGAGTTAGCACTGAGC-3'	pAG416GAL-ccdB-HA (Alberti *et al.* 2007)
**HA-less construct**	
HAless-HindIII_F 5'-GACTAAGCTT(HindIII)TCAGTACTGACAATAAAAAGATTCTTG-3'<br>HAless-HindIII_R 5'-GACTAAGCTT(HindIII)TTA(Stop)GCAGCCCATCACCACTTTG-3'	pYOGM081
**Construct with the *ADH1* promoter**	
ADH1pr_Fwd 5'-TGCGGCCAAGCTCCTGTACAATATGGACTTCCTCTTTTCTG-3'<br>ADH1pr_Rev 5'-GCTTTTTTGTACAAACTTGTGATTGTATATGAGATAGTTGATTG-3'	BY4741 genomic DNA
ADH1pr_pYOGM081_Fwd 5'-CTATCTCATATACAATCACAAGTTTGTACAAAAAAGCTGAACG-3'<br>ADH1pr_pYOGM081_Rev 5'-GAGGAAGTCCATATTGTACAGGAGCTTGGCCGCAAATTAAAG-3'	pYOGM081
**Construct with the *TEF* promoter**	
TEFpr_Fwd 5'-TGCGGCCAAGCTCCTGTGACATGGAGGCCCAGAATACCCTC-3'<br>TEFpr_Rev 5'-GCTTTTTTGTACAAACTTGTGATGGTTGTTTATGTTCGGATGTG-3'	pFA6a-kanMX4 (Wach *et al.* 1994)
TEFpr_pYOGM081_Fwd 5'-CCGAACATAAACAACCATCACAAGTTTGTACAAAAAAGCTGAAC-3'<br>TEFpr_pYOGM081_Rev 5'-CTGGGCCTCCATGTCACAGGAGCTTGGCCGCAAATTAAAGCC-3'	pYOGM081
**Construct with the *CUP1* promoter**	
CUP1pr_Fwd 5'-GCTTTAATTTGCGGCCAAGCTCCTGTAACTTCAACGATTTCTATGATGC-3'<br>CUP1pr_Rev 5'-CAGCTTTTTTGTACAAACTTGTGATTTTATGTGATGATTGATTG-3'	BY4741 genomic DNA
CUP1pr_pYOGM081_Fwd 5'-CAATCATCACATAAAATCACAAGTTTGTACAAAAAAGC TGAACGAGAAACG-3'<br>CUP1pr_pYOGM081_Rev 5'-CATAGAAATCGTTGAAGTTACAGGAGCTTGGCCGCAAA TTAAAGCCTTC-3'	pYOGM081
