# Supplementary material for: The Insertion Green Monster (iGM) Method for Expression of Multiple Exogenous Genes in Yeast
Source: G3 (Bethesda). 2014 Apr 28;4(7):1183–91. doi: 10.1534/g3.114.010868 (PMC4455768; doi:10.1534/g3.114.010868)
Supplement: Supporting Information [file supp_g3.114.010868_TableS2.pdf]

Table S2 List of genotyping primers used in this study.

Deleted ORF	Gene insertion	Primer sequence
YKL069W	TRSP	5'-TAGCGACAGAGTGGTTCAATTC-3'<br>5'-TCAATTTGGCGAACAGGGAATG-3'
YER042W	PSTK	5'-AGTGTTGCAGAATCGAGAAGAG-3'<br>5'-TCATAAATAAGGGCACGTACAC-3'
YOL118C	SECS	5'-ACACATACCAGGATGCTTCTTC-3'<br>5'-GCTGACTAATTTGAAGCTATCG-3'
YLR123C	SPS1	5'-ACAGCCAGAATCATAGACAAAC-3'<br>5'-TTCAGCTGATGTGCCATGTAAC-3'
YER108C	SPS2	5'-GATGTTAAGTCTTTTGCGGCAG-3'<br>5'-AAAGTCGTTGCTGTGAAAATGG-3'
YCL033C	SBP2	5'-AAGAATCCTTGGAGGCTTCAAC-3'<br>5'-GTCCACGATCTCAAACCCTTTC-3'
YKR012C	SBP2L	5'-TTACACAACGCAAACTACGTAC-3'<br>5'-TTAGGACCATCTTGCATTTGAG-3'
YDL242W	EEFSEC	5'-TCAAGCGTTATGTCTTCGACAC-3'<br>5'-GTTTCGATATTCGCACATTTGC-3'
YGL109W	SECP43	5'-ACAAGGAGTTCATGGAACAGAG-3'<br>5'-CAACTAAAGAGTACAACTGTCC-3'
YFR057W	RPL30	5'-CTCTGACATCATTAGAAGCATG-3'<br>5'-AGATAACTCTGAACTGTGCATC-3'
YDL227C	cSPS2	5'-CTCTGTTCCCTCTCATATTTAC-3'<br>5'-CTACTCCAGCATTCTAGTTAAG-3'

SUPPLEMENTARY REFERENCES

Alberti, S., A. D. Gitler, and S. Lindquist, 2007 A suite of Gateway cloning vectors for high-throughput genetic analysis in Saccharomyces cerevisiae. Yeast 24: 913-919.

Goldstein, A. L., and J. H. McCusker, 1999 Three new dominant drug resistance cassettes for gene disruption in Saccharomyces cerevisiae. Yeast 15: 1541-1553.

Sikorski, R. S., and P. Hieter, 1989 A system of shuttle vectors and yeast host strains designed for efficient manipulation of DNA in Saccharomyces cerevisiae. Genetics 122: 19-27.

Wach, A., A. Brachat, R. Pohlmann, and P. Philippsen, 1994 New heterologous modules for classical or PCR-based gene disruptions in Saccharomyces cerevisiae. Yeast 10: 1793-1808.
